# The Immunology of Hormone Receptor Positive Breast Cancer

**DOI:** 10.3389/fimmu.2021.674192

**Published:** 2021-05-11

**Authors:** Jonathan Goldberg, Ricardo G. Pastorello, Tuulia Vallius, Janae Davis, Yvonne Xiaoyong Cui, Judith Agudo, Adrienne G. Waks, Tanya Keenan, Sandra S. McAllister, Sara M. Tolaney, Elizabeth A. Mittendorf, Jennifer L. Guerriero

**Affiliations:** ^1^ Breast Tumor Immunology Laboratory, Department of Cancer Biology, Dana-Farber Cancer Institute, Boston, MA, United States; ^2^ Division of Breast Surgery, Department of Surgery, Brigham and Women’s Hospital, Boston, MA, United States; ^3^ Laboratory of Systems Pharmacology, Department of Systems Biology, Harvard Medical School, Boston, MA, United States; ^4^ Department of Cancer Immunology and Virology, Dana-Farber Cancer Institute, Boston, MA, United States; ^5^ Department of Immunology, Harvard Medical School, Boston, MA, United States; ^6^ Breast Oncology Program, Dana-Farber Cancer Institute, Boston, MA, United States; ^7^ Division of Hematology, Department of Medicine, Brigham and Women’s Hospital, Boston, MA, United States; ^8^ Department of Medicine, Harvard Medical School, Boston, MA, United States; ^9^ Harvard Stem Cell Institute, Cambridge, MA, United States; ^10^ Ludwig Center for Cancer Research at Harvard, Harvard Medical School, Boston, MA, United States

**Keywords:** hormone receptor (HR), breast cancer, immunotherapy, immune exclusion, T-cell exclusion, antigen presentation, clinical trial

## Abstract

Immune checkpoint blockade (ICB) has revolutionized the treatment of cancer patients. The main focus of ICB has been on reinvigorating the adaptive immune response, namely, activating cytotoxic T cells. ICB has demonstrated only modest benefit against advanced breast cancer, as breast tumors typically establish an immune suppressive tumor microenvironment (TME). Triple-negative breast cancer (TNBC) is associated with infiltration of tumor infiltrating lymphocytes (TILs) and patients with TNBC have shown clinical responses to ICB. In contrast, hormone receptor positive (HR+) breast cancer is characterized by low TIL infiltration and minimal response to ICB. Here we review how HR+ breast tumors establish a TME devoid of TILs, have low HLA class I expression, and recruit immune cells, other than T cells, which impact response to therapy. In addition, we review emerging technologies that have been employed to characterize components of the TME to reveal that tumor associated macrophages (TAMs) are abundant in HR+ cancer, are highly immune-suppressive, associated with tumor progression, chemotherapy and ICB-resistance, metastasis and poor survival. We reveal novel therapeutic targets and possible combinations with ICB to enhance anti-tumor immune responses, which may have great potential in HR+ breast cancer.

## Introduction

Immunotherapy represents a paradigm shift in oncology. In particular, immune checkpoint blockade (ICB) has emerged as an efficacious treatment option for many tumor types, providing new therapeutic options for previously untreatable cancers. ICB therapy involves the use of humanized antibodies to target and neutralize immune checkpoint proteins with the goal of invigorating T cell activation and anti-tumor responses. Targeting immune inhibitory molecules, including cytotoxic T-lymphocyte-associated protein 4 (CTLA-4), programmed death-1 (PD-1) and its ligand, PD-L1, aims to reinvigorate exhausted T cells, thus enabling improved tumor antigen recognition and cytotoxic activity (1). The benefits of ICB, however, are not equally realized among different cancer types. In general, cancers that respond to ICB have at least one of these three key features: high tumor mutational burden (TMB), high numbers of tumor-infiltrating-lymphocytes (TILs) and/or high PD-L1 expression (2). Tumors from melanoma and lung cancer patients generally exhibit all of these features and have demonstrated superior responses to ICB (3–5). In contrast, breast tumors generally have low TMB, are often poorly infiltrated by TILs, have low levels of PD-L1 expression, and are thus considered to be nonimmunogenic and less responsive to ICB (6–9).

Breast cancer is a histopathologically and molecularly heterogeneous disease, ranging from the more indolent luminal A tumors, which are generally estrogen receptor positive (ER+) and human epidermal growth factor receptor 2 (HER2)-negative, to the highly aggressive, basal-like triple-negative tumors, which are negative for the ER, progesterone receptor (PR) and HER2. Triple-negative breast cancer (TNBC) generally has a higher mutational load, greater TIL infiltrate and higher PD-L1 expression relative to other breast cancer subtypes (10–12). Consistent with those immune features, the greatest successes reported to date of ICB in breast cancer clinical trials have been in patients with TNBC. However, chemotherapy combinations may prove effective, particularly for breast cancers that are not innately sensitive to ICB (13). Two agents, the anti-PD-L1 agent atezolizumab and the anti-PD-1 agent pembrolizumab, have been approved for use in combination with nab-paclitaxel or chemotherapy, respectively, for the treatment of metastatic TNBC following the results of phase 3 clinical trials showing improvement in progression free survival (PFS) with the use of these agents (14–16). Importantly, atezolizumab and nab-paclitaxel also led to a clinically meaningful improvement in overall survival (OS) in patients with PD-L1 positive disease (15). To date ICB has not been approved for the treatment of other subtypes of breast cancer. Given that TNBC comprises only 15% of all breast cancer cases (17), there is an urgent need to better understand the underlying basis of diminished immune responses to these other subtypes, with the goal of making those subtypes susceptible to ICB or other agents that act by enhancing anti-tumor immune responses.

Hormone receptor positive (HR+) breast cancer comprises approximately 70% of breast cancers and is characterized by dependence on ER signaling (17). HR+ breast cancer is generally a more indolent breast cancer subtype, has a low TMB and low PD-L1 expression (9, 18). Importantly, among the different breast cancer subtypes, HR+ tumors tend to have the lowest numbers of TILs (8, 15, 19–25). There are currently no FDA approved ICB agents for the treatment of HR+ breast cancer, however in the past several years new evidence has emerged showing immunogenic subsets of HR+ tumors (26) and that ICB might be effective in combination with the right chemotherapy (13). In addition, advances in single cell sequencing and imaging technologies have revealed a wide diversity of both immune and non-immune cells that comprise the HR+ TME. Nevertheless, with respect to HR+ breast cancer, there remains a gap in knowledge as to how baseline immune contexture affects a patient’s prognosis and how individualized treatments can be developed based on the characteristics of a patient’s TME. In this review, we summarize what is known about the immunogenicity of HR+ breast cancer and the opportunities to target HR+ tumors with ICB and other immune-activating therapies.

## The Role of Tumor-Infiltrating Lymphocytes (TILs) in HR+ Breast Cancer

Lymphocytes, which are white blood cells including T cells, B cells and natural killer cells, were first correlated with breast cancer outcome in the early 1990s (27). Since then TILs have been studied extensively in breast cancer and have been shown to have both prognostic and predictive value (28), yet their role in HR+ breast cancer is more elusive (**Table 1**). TIL analysis in clinical laboratories is performed using a continuous parameter on a single hematoxylin and eosin (H&E)-stained tumor section and criteria described by Denkert et al. is used to score infiltrating TILs (40). Intratumoral TILs (iTILs) are defined as intraepithelial mononuclear cells within tumor cell nests or in direct contact with tumor cells, and stromal TILs (sTILs) as lymphocytes in the tumor stroma without direct contact with tumor cells. While stromal and iTILs are generally correlated, iTILS are far less abundant and more difficult to identify on H&E sections and new guidelines advocate to quantify only sTILs on H&E-stained tumor sections (54). Interestingly, a study conducted by the International Immuno-Oncology Biomarker Working Group demonstrated that a software-guided image evaluation approach could improve inter-observer variability (55). These efforts have focused on standardizing an approach to establish TILs as a predictive and prognostic biomarker to guide the clinical management of breast cancer. However, as described in **Table 1** there are different methods of TIL assessment that has been reported which may account for differences observed between studies.

**Table 1:** Summary of the association of tumor infiltrating lymphocytes (TILs) in HR+ breast cancer with clinical outcome.

Publication	Study Design (Trial Name)	Number of Evaluable HR+ Samples	TIL Assay	Findings
**Adjuvant Setting**				
Loi et al. (19)	Prospectively defined, retrospective (BIG 02-98 trial)	1078	sTILs, iTILs in H&E	No association found between TILs and DFS or TILs and OS
Loi et al. (29)	Prospectively defined, retrospective (FinHER trial)	694	sTILs, iTILs in H&E	No association found between TILs and distant DFS or TILs and OS
Dieci et al. (30)	Prospectively defined, retrospective (Two French multicentric trials)	501	sTILs, iTILs in H&E	No association found between TILs and OS
Carbognin et al. (31)	Sensitivity analysis of data from Loi et al. (19, 29) & Dieci et al. (30)	2132	sTILs, iTILs in H&E	No association found between TILs and OS
Krishnamurti et al. (32)	Retrospective, archival tissues	187	sTILs in H&E	Negative association found between TILs and Oncotype DX recurrence score
Miyoshi et al. (33)	Retrospective, multicentric	639	sTILs in H&E	No association found between TILs and timing of recurrence
Fujimoto et al. (34)	Retrospective, archival tissues	519	sTILs, iTILs in H&E	Ki67-low group: high-TILs showed significant unfavorable DFS Ki67-high group: high-TILs showed nonsignificant favorable DFS
Ali et al. (35)	Prospectively defined, retrospective (SEARCH, BCCA, NBCS, NEAT trials)	6714	IHC staining for CD8+ and FOXP3+ sTILs and iTILs	Intratumoral CD8+ lymphocytes not associated with outcome
Sobral-Leite et al. (36)	Prospectively defined, retrospective (IKA trial)	563	IHC staining for CD4, CD8, and FOXP3	High CD8+ T cell infiltration associated with increased risk of recurrence
Gu-Trantien et al. (37)	Retrospective (fresh and archival tissues)	510	Gene expression	An 8-gene Tfh signature showed significant prognostic values in luminal tumors
Liu et al. (38)	Retrospective, archival tissues	2351	IHC staining for FOXP3+ sTILs and iTILs	FOXP3+ regulatory T cells were associated with poor prognosis
Koletsa et al. (39)	Prospectively defined, retrospective (HE10/97, HE10/00 trials)	600	IHC staining for CD3+, CD8+ and FOXP3+ sTILs, iTILs and total TILs	Assessment of CD3+, CD8+ and FOXP3+ lymphocytes densities adds no value over a traditional stromal TILs assment
**Post-neoadjuvant Setting**			
Denkert et al. (40)	Prospectively defined, retrospective (GeparDuo and GeparTrio trials)	659	sTILs, iTILs in H&E, immune mRNA markers	Increased TILs associated with pCR
Issa-Nummer et al. (41)	Prospectively defined, retrospective (PREDICT trial)	209	sTILs, iTILs in H&E	Validation of results in Denkert et al. (40)
Denkert et al. (10)	Prospectively defined, retrospective (GeparDuo, GeparTrio, GeparQuattro, GeparQuinto, GeparSixto, and GeparSepto trials)	832	sTILs in H&E	Increased TILs associated with shorter OS
Skriver et al. (42)	Prospectively defined, retrospective (phase II Danish Breast Cancer Group trial)	106	sTILs in H&E	Increased TILs from baseline associated with poor treatment response
Ono et al. (43)	Retrospective, archival tissues	46	sTILs in H&E	No correlation found between TILs and pCR
Hwang et al. (44)	Retrospective, archival tissues	131	sTILs in H&E	No correlation found between TILs and pCR
Russo et al. (45)	Retrospective, archival tissues	119	sTILs in H&E	No correlation between TILs and survival
Ali et al. (46)	Prospectively defined, retrospective (ARTemis trial)	446	computational pathology of sTILs in H&E	Lymphocyte density associated with pCR in multivariate analysis
Seo et al. (47)	Retrospective, archival tissues	100	IHC staining for CD4+, CD8+ and FOXP3+ sTILs and iTILs	CD8+ TILs were independent predictors for pCR
Brown et al. (48)	Retrospective, archival tissues	58	Quantitative IF for CD3, CD8, and CD20 sTILs	CD20+, but not CD3+ or CD8+ lymphocytes predict pCR
**Post-neoadjuvant Setting**				
Watanabe et al. (49)	Retrospective, archival tissues	Pre-Tx: 91 Post-Tx: 80	iTILs in H&E	Low TILs associated with improved RFS only in post-Tx group
Pelekanou et al. (50)	Retrospective, archival tissues	46	sTILs in H&E	Increased TILs post-Tx associated with longer 5-year RFS
Hamy et al. (51)	Retrospective, archival tissues	223	sTILs in H&E	No association between TILs and DFS
Ladoire et al. (52)	Retrospective, archival tissues	88	IHC staining for CD8+ and FOXP3+ sTILs	High CD8+ and low FOXP3+ lymphocyte infiltrates associated with improved RFS and OS
Asano et al. (53)	Retrospective, archival tissues	80	sTILs in H&E	RCB-TILs score predicts recurrence, may be a more sensitive indicator than TILs alone

sTIL, stromal tumor infiltrating lymphocytes; iTIL intratumoral tumor infiltrating lymphocytes; H&E, hematoxylin and eosin; DFS, disease free survival; OS, overall survival; pCR, pathological complete response.

### TILs in Breast Tumors Before and After Neoadjuvant Systemic Therapy

Over a decade ago, it was shown that the presence of TILs is an independent predictor of response to neoadjuvant chemotherapy (NAC) in all subsets of breast cancer, where high levels of TILs were associated with increased pathological complete response (pCR) rates compared to tumors that demonstrated absence of TILs (40). Subsequently, tumors from the BIG 02-98 trial revealed that TILs are associated with clinical benefit from adjuvant chemotherapy in patients with TNBC and HER2-positive (HER2+) breast cancer (19). In addition, this trial demonstrated that TILs were significantly lower in HR+/HER2- tumors compared to other subtypes (19). The extent of clinical response to NAC is a prognostic factor for TNBC, HR+ and HER2+ breast cancer, with the best clinical outcomes seen in patients that experience a pCR (56, 57). A meta-analysis of six randomized trials by the German Breast Group showed that increased TILs were predictive for more favorable response to NAC for all breast cancer subtypes, where higher pCR rates were observed when tumors were categorized as high TILs. In this study, TILs were analyzed as predefined groups of low (0-10% immune cells in stromal tissue within the tumor), intermediate (11-59%), and high TILs (≥60%). A univariable analysis revealed that a 10% increase in TILs was associated with longer DFS in TNBC and HER2+ breast cancer but not in luminal-HER2- tumors. Interestingly, an increase in TILs was associated with longer OS in TNBC, had no association with HER2+ breast cancer and was associated with shorter survival in luminal-HER2- tumors (10). The finding that TILs have a positive short-term prognostic value (as measured by response at surgery) whereas they have a negative long-term prognostic value highlights the complexity of TILs in the TME. Previous work by this same group had shown the positive association with short-term responses in HR+ breast cancer and these findings were confirmed by the same group in the PREDICT study; and should be noted that TNBC was associated with higher TILs compared to HR+ breast cancer (10, 41, 58, 59). In another study evaluating baseline biopsies prior to chemotherapy, where both areas of stroma infiltrated by lymphocytes (proportional score) and intensity of lymphatic infiltration (intensity score) were taken into consideration, high TILs score was associated with pCR in TNBC but not for Her2+ or HR+ tumors (43). Other retrospective cohorts evaluating pre-NAC TILs association with pCR similarly failed to find a significant correlation, most likely due to the limited number of HR+ tumors used in pCR prediction (44, 45, 60). The significance of TILs in patients treated with neoadjuvant hormonal therapy has been recently studied as part of a nationwide phase II trial conducted by the Danish Breast Cancer Group. The group evaluated pretreatment core biopsies and surgical specimens for percentage of TILs and pathological complete response was assessed using Residual Cancer Burden (RCB) index. The group reported that increasing TILs during letrozole treatment was significantly associated with a poor treatment response (42). Interestingly they propose that an increase in TILs during endocrine therapy might imply immunogenicity, and these patients could be targetable by immunotherapy (42).

Unconventional approaches to measure lymphocyte infiltration have also revealed interesting results from analysis of baseline tumors. In a cohort of TNBC patients, stromal TILs and TILs measured by tumor infiltrating lymphocyte volume (TILV) were significantly correlated with pCR (61). In that study TILV were calculated using the formula TILV = % stroma in tumor x % stromal TILs; where stromal TILs were assessed according to the standardization and guidelines of the international TILs working group (54). In an analysis of the ARTemis trial using computational pathology, lymphocyte density was significantly associated with pCR in multivariate analysis but there was no association between pre-treatment lymphocyte density and survival in either HR+ or HR- patients treated with NAC (46). Subset analyses of lymphocyte infiltrates have been described in breast cancer where TILs are largely composed of CD4+ and CD8+ T cells (62). In a retrospective study, CD8+ TILs in pre-chemotherapeutic biopsy specimens were found to be independent predictors for pCR irrespective of breast cancer subtype (47). Conversely, in another study, CD20+ lymphocytes (generally thought to be B cells) scored by quantitative immunofluorescence positively predicted pCR in response to NAC irrespective of HR and HER2 status, whereas CD3+ and CD8+ lymphocytes did not (48).

In addition to the value of TILs as a potential biomarker predicting response to NAC, there is an interest from the International Immuno-Oncology Biomarker Working Group on Breast Cancer (IIBWG) in evaluating the utility of TILs to refine risk stratification in patients with residual disease following neoadjuvant treatment (63). A retrospective multicenter study with TNBC patients concluded that the presence of TILs in residual disease following NAC was a strong favorable prognostic factor for both metastasis-free and OS in this subtype of breast cancer (64); less work has been done in HR+ disease. Watanabe and colleagues evaluated TILs in HR+/HER2- primary breast cancers before and after NAC, and concluded that low TILs following NAC, but not at baseline, were associated with a significantly better recurrence free survival (RFS) (49). In another cohort that included all breast cancer subtypes, increased TIL infiltration after NAC compared to baseline was associated with longer 5-year RFS (50). Furthermore, Ladoire and colleagues found the association of both high CD8+ and low FOXP3+ lymphocyte infiltrates following NAC was linked with improved RFS and OS in a cohort that included all breast cancer subtypes (52). In addition, a combined score associating CD8/FOXP3 ratio and pathological AJCC staging isolated a subgroup of patients with a long-term overall survival of 100% (52). In contrast, in a retrospective French cohort, high post-NAC TILs were associated with worse disease-free survival (DFS) in HER2+ patients, but not in TNBC and HR+ patients (51). Asano and colleagues (53), combined the residual cancer burden (RCB) index (57) and TILs (“RCB-TILs”) to predict survival after NAC. In their multivariate analysis, RCB-TILs was an independent factor for recurrence overall and within each of the breast cancer subtypes, suggesting RCB-TILs may be a more sensitive prognostic marker than TILs or RCB alone (53). The IIBWG has recently launched an international effort to include TILs in a new version of the RCB index to better stratify patients post-NAC (63).

### TILs in HR+ Breast Tumors Managed With Adjuvant Systemic Therapy

Studies that have evaluated TILs in early-stage treatment-naïve breast tumors managed with adjuvant systemic therapy have so far generally failed to demonstrate prognostic value in HR+ tumors (19, 29, 32, 65, 66). In the BIG 02-98 trial, in which patients were randomized to a doxorubicin-based regimen with or without docetaxel, TILs were not significantly associated with DFS or OS in HR+/HER2- (19). These findings were confirmed in HR+/HER2- cases from the FinHER trial, in which patients were randomized to adjuvant docetaxel or vinorelbine regimens, followed by fluorouracil, epirubicin and cyclophosphamide (29). Similarly, by combining patients from two French multicentric trials, randomized by addition of adjuvant anthracycline-based therapy, a significant association between TILs and OS was not identified in HR+/HER2- breast tumors (30). The aforementioned studies were included in a sensitivity analysis of randomized trials in the adjuvant and neoadjuvant setting, which confirmed there was no association between baseline TILs and OS for HR+/HER2- tumors (31). In a more recent study, patients who underwent mastectomy without neoadjuvant treatments were evaluated for TILs. In HR+ breast cancer, there was a negative association between Oncotype DX recurrence score and both overall and peripheral TILs, where peripheral TILs were evaluated as the percentage of stromal lymphocytes encountered in the entire circumferential invasive tumor front. The negative association between TILs and Oncotype DX score may indicate the possible prognostic value of TILs in HR+ breast cancer. However, peripheral TILs were significantly associated with OS and DFS in TNBC but not in HR+ breast cancer (32).

It is noteworthy that several studies have identified a link between subpopulations of T cells in HR+ breast tumors and long-term outcomes following adjuvant systemic therapy. In Ki67-high breast cancers, high TILs were associated with favorable DFS, irrespective of subtype, but increasing TIL levels correlated with worse DFS in the Ki67-low group (defined as ≤ 25%) with the HR+/HER2- subtype. These results highlight variation in TIL prognostic significance between Ki67-high and -low breast cancers, particularly for the HR+/HER2- subtype (34). In a large study of 12,439 patients, assessment of T cell infiltration in breast cancer indicated that intratumoral CD8+ lymphocytes were associated with worse outcomes in HR+/HER2- patients, however the association did not remain significant in multivariate analysis (35). Similarly, in a retrospective analysis of a prospective randomized trial in HR+ breast cancer in which postmenopausal patients with early stage HR+/HER2- breast cancer were randomized to tamoxifen treatment or no adjuvant therapy, it was found that tumors with high CD8+ T cell infiltrates were associated with increased recurrence risk (36). Other T cell subsets have also been examined in early-stage HR+ breast tumors treated with adjuvant systemic therapy. To better understand CD4+ follicular helper T cells (Tfh), an 8-gene Tfh signature was reported, which was consistently prognostic in luminal tumors, as well as in other subtypes (37). Conversely, FOXP3+ regulatory T cells assessed in treatment naïve tumors were shown to be an indicator of poor prognosis in HR+ breast cancer, but of favorable prognosis in HR-/HER2+ tumors (38). However, recent work including all subtypes of breast carcinomas concluded that CD3+, CD8+ and FOXP3+ lymphocyte densities did not add prognostic information over stromal TILs assessed on H&E in early intermediate/high-risk breast cancer treated with adjuvant chemotherapy (39). These findings confirm the complexity of the TME in HR+ breast cancer and taken together, indicate that further investigation is necessary to determine the predictive and prognostic values of TILs in HR+ breast cancer.

### Location of TILs in Breast Tumors

The location and organization of TILs, in particular, T cells in tumors may be important in their ability to become activated and exert anti-tumor effects as well as B cells in tertiary lymphoid structures (reviewed in the next section). Important work has been done to investigate the spatial location of T cells in TNBC which led to a tumor immune microenvironment (TIME) classification to group tumors into patterns according to CD8^+^ TIL spatial distribution (67). Immunoreactive TMEs were identified that consisted of tumoral infiltration of granzyme B+CD8+ T cells (GzmB+CD8+ T cells), a type 1 IFN signature, and elevated expression of immune inhibitory molecules such as indoleamine 2,3-dioxygenase (IDO) and PD-L1, which correlated with favorable clinical outcomes. This same group showed that “immune-cold” TMEs, which had absence of tumoral CD8+ T cells, were defined by elevated expression of the immunosuppressive marker B7-H4, signatures of fibrotic stroma and poor outcomes (67). Interestingly, a significant accumulation of proinflammatory CD68+CD206- macrophages were found in tumors with high infiltration of CD8+ T cells compared to TNBC with less CD8+ T cells (67). Indeed, localization and composition of T-cells in TNBC has demonstrated that the immunomodulatory subtypes are associated with the highest expression of adaptive immune-related gene signatures and a fully inflamed spatial pattern (68). Other work in TNBC has focused on exclusion of T cells from tumor cell clusters and spatial-profile analysis and mathematical modeling suggests a possible inhibitory signal inside tumor cell clusters, which prevents CD8+ T cells from infiltrating into tumor cell clusters (69). The location of T cells may help understand responses to ICB and identify tumors with high likelihood of response in TNBC and may extend to HR+ breast cancer. However, characterization of the spatial organization of T cells and other immune cells in HR+ breast cancer remains an unmet need.

### Tumor-Associated Tertiary Lymphoid Structures (TLS)

TLS are ectopic lymphoid organs, composed of lymphoid cells that arise in chronic inflammatory states, including tumors (70). These structures have considerable morphological overlap with secondary lymphoid organs (SLO), particularly lymph nodes, although “TLS” can refer to structures of varying complexities, from simple lymphocytic clusters to elaborate formations highly reminiscent of a SLO (71). TLS exhibit characteristics of structures in the lymph nodes associated with the generation of an adaptive immune response, including a T cell zone with mature dendritic cells, a germinal center with follicular dendritic cells and proliferating B cells, and high endothelial venules (72). There is increasing interest in studying tumor-associated TLS as recent work has revealed these structures to be valuable biomarkers in multiple tumor types, including breast cancer (73–78). The presence of TLS structures has demonstrated both prognostic and predictive value in breast carcinomas, although data is discrepant on whether these structures are associated with favorable or detrimental outcomes. Martinet and colleagues found that high densities of tumor-associated high endothelial venules, a common constituent of TLSs, were independently associated with longer DFS and OS in breast cancer patients, irrespective of HR status (79). In a study conducted by Liu and colleagues, TLSs were significantly associated with favorable DFS in patients with HER2+ breast cancer, independent of TIL status (76). In contrast, a recent analysis of all breast cancer subtypes reported that the presence and density of peritumoral TLSs were not independently associated with DFS and OS (80). Interestingly, TLSs have been demonstrated to be significant predictors of pCR in TNBC patients treated with NAC (78). It is important to note that tumor-associated TLS assessment has not been standardized, although it should preferably be performed in full-face sections, as biopsies or tissue microarrays likely cannot accurately reflect TLS status (81). In addition, H&E evaluation underestimates the presence of these structures compared to immunohistochemistry (IHC). Using IHC, one group has identified TLS by staining for CD45 to identify leukocytes and CD20/CD3 to identify B cell follicles surrounded/adjacent to T cell zones, respectively. The study revealed that and intra- and inter-observer agreement is superior using IHC compared to H&E (81). Modern multiplex imaging technologies are emerging as an improved modality to study these structures as evident in several recent publications (73, 77, 82).

### Antigen Presentation in HR+ Breast Cancer

As we discussed above, the number of infiltrating TILs within a breast tumor has both prognostic and predictive implications. In order for anti-tumor T cell responses to be generated, tumor antigens must be presented complexed with human leukocyte antigen (HLA) molecules at the cell surface for recognition by T cells (83). Studies have shown that HLA downregulation is an important mechanism of immune evasion that has been observed in multiple tumor types (84–86). The true frequency of HLA downregulation in cancer is controversial, in part due to differing antibodies used to detect HLA-class I (HLA-I) molecules. In the past decade, the EMR8-5 antibody has emerged as the method of choice to detect surface HLA-I expression on tumor cells (87–89). Torigoe and colleagues used the EMR8-5 antibody by IHC to assess the frequency of HLA class I downregulation in various cancer tissues (n=246). Using criteria established by the HLA and Cancer Component of the 12th International Histocompatibility Workshop (90), HLA expression was scored based on cell expression and intensity. The group found that HLA-I was decreased in 20-42% of lung, liver, colon, renal and urothelial cancer cases (91) whereas 85% of breast cancer cases had loss of or decreased HLA-I expression (91). Another report from Kaneko and colleagues reported HLA-I downregulation in 32.5% of breast tumors and was significantly associated with worse clinical features (nodal involvement and stage) as well as worse disease-free interval (84). Similarly, using multiple antibodies against HLA, Garrido and colleagues revealed various types of HLA-I alterations in 79 of 98 (81%) of breast tumors, including complete HLA-I loss in 53 (54%) of the samples (92). HLA-I downregulation may be particularly important in HR+ breast cancer as Sinn and colleagues measured HLA-I expression in 863 breast cancer cases from the GeparTrio trial, including all subtypes of breast cancer. The group found that HR+/HER2- cancers had the lowest level of HLA class I expression compared to other subtypes (93). Furthermore, a negative correlation between mRNA expression of the estrogen receptor 1 (ESR1) gene and HLA was also found in the Cancer Cell Line Encyclopedia (CCLE) (94). Importantly, in a study of The Cancer Genome Atlas (TCGA), ESR1 expression was found to be inversely correlated with HLA-A and CD8B gene expression (94). These studies suggest that HLA expression may be inversely correlated with ER expression and positively correlated with T cell infiltration. However, the mechanism underlying this relationship has not yet been elucidated (95, 96). Taken together these data suggest that HLA downregulation may be an important mechanism of immune evasion in breast cancer and in particular in HR+ breast cancer.

Prior to presentation of antigen complexed with an HLA molecule, that antigen must undergo processing. Components of the antigen-processing machinery (APM) have also been evaluated in breast cancer. Liu and colleagues found differential expression of antigen-processing molecules between primary breast tumors with and without associated brain metastases (n=65, 49 HR+) (96). In particular, primary breast lesions in patients who later developed brain metastases showed lower beta 2 microglobulin (B2M; the co-receptor for HLA) expression as well as other APM components, such as transporter associated with antigen processing 1 and 2 (TAP1/2), and calnexin, which are essential components for antigen processing and loading on HLA. In addition, CD8 T cell infiltration was significantly higher in primary breast lesions without an associated brain metastasis and was correlated with TAP1 expression. Preclinical data further support these findings. Murine tumor cells stably transfected with silencing hairpin (sh)RNA for TAP1 demonstrated a decreased susceptibility to cytotoxic T lymphocytes *in vitro* and an increased frequency of spontaneous brain metastasis *in vivo* (96). These data suggest that a deficiency in antigen-processing machinery may increase the likelihood of metastasis through deficient immune surveillance.

The value of HLA downregulation as a biomarker in breast cancer has been assessed in several studies of early-stage and metastatic disease. Although the data are conflicting, the majority of studies indicate that HLA-I downregulation is associated with poor prognosis. In a large retrospective study, the correlation of HLA-I expression with clinical outcome was assessed in 465 surgically resected breast cancer specimens including 310 primary HR+ tumors (97). Complete loss of HLA-I was observed in about 18% of both the HR+ and HR- subsets and survival analysis revealed that HLA-I expression loss was significantly correlated with worse disease-specific survival (DSS). In addition, HLA-I was found to be an independent prognostic factor for adverse DSS in patients with stage II-IV breast cancer. Interestingly, in contrast to the previously mentioned studies, in a study of 439 invasive primary breast cancers including all subtypes, Madjd and colleagues found strong HLA-I staining correlated with the development of metastasis and HLA-I downregulation to be associated with improved clinical outcomes (98).

### Natural Killer Cells in HR+ Breast Cancer

While low expression of MHC-I may limit CD8 T cell recognition and response to HR+ breast tumors, the lack of MHC-I molecules should in turn promote NK cell activation, representing an alternate immunotherapeutic target (99–101). In general, NK cells account for a small portion of infiltrating lymphocytes in breast tumors (102, 103). Interestingly, analyses of TCGA and METABRIC samples revealed HR+ tumors have lower NK cell gene expression compared to TNBC tumors and immune-rich HR+ tumors have a lower proportions of NK cells compared to immune-rich TNBC tumors (104, 105). The combination of MHC-I downregulation and NK cell exclusion has not been analyzed in the literature and is an active line of investigation in our lab. Although NK cell infiltration is limited in HR+ tumors, HR+ breast cancer cell lines are more susceptible to IL-2 stimulated NK cell lysis than are TNBC or HER2+ cell lines (106–108), indicating that potential strategies to target HR+ tumors may include adoptive transfer of exogenously stimulated or genetically altered NK cells. Multiple pre-clinical investigations showed efficacy of NK-CAR cells targeting HER2 in HER2+ breast cancer (109, 110), tissue-factor in TNBC (111), epithelial cell adhesion molecule (EpCAM) in both HER2+ and TNBC (112) and epidermal growth factor (EGFR) in all breast cancer subtypes (113). Importantly, EpCAM is highly expressed in all breast cancer subtypes and thus can serve as a potential NK-CAR target in HR+ tumors (114). Overall, the majority of NK cell-based immunotherapy investigations have centered around HER2+ breast cancer as HER2-targeting monoclonal antibodies work, in part, through antibody-dependent cellular cytotoxicity, of which NK cells play a crucial role (115). NK cell immunotherapy has gained traction in TNBC, including a phase 1 investigation of PD-1 inhibition in combination with a novel inhibitor of the NK cell checkpoint poliovirus receptor related immunoglobulin domain containing (PVRIG) (NCT03667716). Given that HR+ tumors have low MHC-I and HR+ cell lines are highly susceptible to NK cell cytotoxicity, there may be great opportunity for NK cell-based therapy in HR+ breast cancer and further pre-clinical and clinical investigations are warranted.

### Beyond TILs: Tumor Associated Macrophages (TAMs) in the TME

Historically, HR+ breast tumors have been considered immunologically cold as there are relatively few T cells associated with these tumors (26). However, other immune cells are associated with the TME in breast cancer. Beyond T cell subsets (cytotoxic T cells, T regulatory T cells), and other lymphocytes (natural killer cells and B cells), myeloid cells (macrophages and dendritic cells), plasmacytoid dendritic cells, and neutrophils have been identified in breast tumors, all of which are known to play critical roles in immunomodulation of cancer progression (116). In an analysis of 11,000 HR+ breast tumors, the immune cell type that correlated most significantly with poor clinical outcome was the presence of TAMs (117, 118). TAMs are a heterogeneous population of cells, generally characterized into an M2/M1 phenotypic and functional dichotomy, although TAMS are phenotypically much more dynamic and diverse. “M2-like” macrophages promote tissue remodeling and repair, secrete anti-inflammatory cytokines, and attract T regulatory and Th2 T cell subsets devoid of cytotoxic functions. TAMs are generally more “M2-like” and show pro-tumor functions by promoting tumor survival, proliferation, angiogenesis, and dissemination (119–125). Alternatively, “M1-like” macrophages are potent effector cells that kill microorganisms and tumor cells and can recruit cytotoxic T lymphocytes (CTLs) to activate adaptive immune responses. They can mediate phagocytosis and cross presentation of antigen to T cells. Clinically, the presence of TAMs is associated with metastasis (119) and poor survival (120, 121, 124), and has been shown to induce endocrine resistance in HR+ breast cancer cells *in vitro* and *in vivo* through NF-κB and IL-6-dependent signaling pathways (126). Importantly, a higher fraction of “M1”-like TAMs in HR+ breast cancer correlated with a higher pCR rate as well as prolonged DFS and OS (118). We recently reported that in HR+/HER2- breast tumors analyzed before and after NAC, sTIL and CD8+ cells were significantly decreased after treatment, whereas expression analyses revealed that there was increased expression of immunosuppressive (M2-like) macrophage-specific genes after chemotherapy. Macrophage biology and mechanisms of immune suppression in breast cancer has been recently reviewed by Mehta and colleagues (127). Macrophage reprogramming has shown tolerability and promise in solid tumors including breast cancer (128), and has been recently reviewed by Mehta and colleagues (127). Further work to identify strategies to harness the anti-tumor potential of macrophages may offer potential opportunities for the treatment of HR+ breast cancer.

## Immune Checkpoints and Immunotherapy Trials in HR+ Breast Cancer

The first clinical target of ICB therapy was the T cell inhibitory molecule, cytotoxic T-lymphocyte-associated protein 4 (CTLA-4; CD152) (129–131). Subsequently, ICB agents targeting the T cell inhibitory molecule, programmed cell death protein 1 (PD-1; CD279) (132), and its ligand, PD-L1 (CD247) (133), were developed for the clinic. PD-1 is a receptor expressed mainly by T cells. Its ligand, PD-L1, is a transmembrane protein that plays a crucial role in shutting down active T cell responses and can be expressed on both tumor and immune cells (12, 134). PD-L1 binding to PD-1 functions as an adaptive mechanism for T cell inhibition, and in the context of cancer, induces tumor immune-suppression (2, 135). Sobral-Leite and colleagues characterized PD-L1 expression in 410 primary, treatment-naïve, breast tumors (162 HR+/HER2-, 101 HER2+ and 147 TNBC). PD-L1 positivity was defined as > 1% of immune or tumor cells as assessed by the E1L3N antibody clone. HR+/HER2- tumors had the lowest TIL density and PD-L1 expression. PD-L1-positivity was observed in 53.1% of HR+/HER2-, 73.3% of HER2+, and 84.4% of TNBC tumors and PD-L1 expression showed a strong correlation with TIL density (25). Similar to TILs, PD-L1 expression has been found to be a prognostic marker in breast cancer, with studies demonstrating an association between PD-L1 expression and improved prognosis in TNBC but not HR+ breast cancer (25, 136–138). There are some data however, indicating that PD-L1 gene expression is associated with improved distant metastasis-free interval, progression-free interval and overall survival in HR+/HER2- breast cancer. In a recent study of 562 breast tumors, PD-L1 protein and gene expression was shown to be associated with a favorable prognosis in early stage invasive HR+/HER2- breast cancer (139). In addition, PD-L1 gene expression added prognostic value to currently validated 21- and 70-gene expression signatures in the same cohort as well as in an additional cohort of 1,081 patients (139).

Despite the limited number of TILs, low PD-L1 expression and low mutational burden in HR+ breast cancer (20), there has been an effort to determine if ICB has a role in HR+ disease (**Table 2**). While, to date, clinical trials testing ICB in HR+ breast cancer have not yet translated to FDA approval, there is opportunity to learn from both past and ongoing trials to identify the ideal therapeutic sequencing, combination strategies and patient population to extract value in this “immunologically cold” subtype of breast cancer, as reviewed below.

**Table 2:** Clinical trials in HR+ breast cancer assessing the safety and efficacy of ICB as monotherapy or in combination with chemotherapy and other treatment modalities.

Trial Identifier (name)	Treatments	Number of Evaluable HR+ Samples	Patient Population	Phase	Results
**Monotherapy**					
NCT02054806 (KEYNOTE-028)	Pembrolizumab	25	Metastatic PDL1+ Heavily pretreated	1b	ORR of 12% (3/25), CBR was 20% with a median duration of response of 12 months
NCT01772004 (JAVELIN)	Avelumab	72	Metastatic Heavily pretreated	1b	ORR of 2.8% (2/72). Lack of response was irrespective of PDL1 status
**Chemotherapy Combination**					
NCT03051659	Pembrolizumab Eribulin	44	Metastatic Moderately pretreated	II	Addition of Pembrolizumab did not effect mPFS (4.1 *vs* 4.2). No trend with PDL1 status, TILs, TMB. Grade 3-4 AEs seen in 54.6% of patients
NCT03044730	Pembrolizumab Capecitabine	14 (16 TNBC)	Metastatic Endocrine resistant	II	Of the 29 evaluable patients, ORR was 14%, CBR was 28% with a median PFS of 4 months. The response rates did not differ between subtypes
NCT01042379 (ISPY-2)	Pembrolizumab Paclitaxel Doxorubicin Cyclophosphamide	40	Neoadjuvant	II	Addition of Pembrolizumab nearly tripled the PFS (34% *vs* 13%). Likelihood of success in a phase III trial prediction was 99.6%
**Other Systemic Therapies**					
NCT01042379 (ISPY-2)	Durvalumab Olaparib Paclitaxel	52	NeoadjuvantBRCA+/-	II	Estimated pCR of 28% with a 74.5% likelihood of success in a stage III trial
NCT02734004 (MEDIOLA)	Durvalumab Olaparib	13 (21 TNBC)	Metastatic	II	Of the 30 evaluable patients, at 12 weeks, DCR was 50% with a median PFS of 8.2 months. Survival by subtype was comparable
NCT02779751 (JPCE)	Pembrolizumab Abemaciclib	28	Metastatic Endocrine resistant	Ib	At 12 months, ORR was 28%, DCR was 82% with a median PFS of 8.9 months.
**Radiotherapy**					
NCT03051672	Pembrolizumab Radiotherapy	18	Metastatic	II	No objective response observed

### ICB Monotherapy in HR+ Breast Cancer

The first trials evaluating ICB as monotherapy in metastatic HR+ disease resulted in only modest response rates. For example, in the KEYNOTE-028 phase 1b trial, 25 heavily pretreated patients with metastatic, PD-L1+, HR+/HER2- breast cancer were administered pembrolizumab monotherapy (140). The objective response rate (ORR) in this cohort was 12% (partial response (PR; n=3), complete response (CR; n=0) with a clinical benefit rate [defined as CR, PR, or stable disease (SD) ≥ 24 weeks] of 20%. The median duration of response reached 12 months, which was higher than expected in this cohort of patients who were chemotherapy and/or endocrine therapy resistant. Of note, two of the three responders had a histology of invasive lobular carcinoma. The ORR in the HR+ cohort (ORR = 12%) was lower than that found for PD-L1+ TNBC patients (ORR = 18.5%) in the KEYNOTE-012 study (141), suggesting this treatment strategy may be more effective in a subset of patients with TNBC. Interestingly, the variation in PD-L1 expression between TNBC and HR+/HER2- breast cancer was also evident in screening participants for the KEYNOTE-012 TNBC study, in which 59% of the total screened had PD-L1+ tumors (141) compared to the KEYNOTE-028 HR+/HER2- study, where only 19% of the total screened were PD-L1+ (140). It is worth noting that not all PD-L1+ TNBC patients derive benefit from ICB and additional work is warranted for novel biomarkers that can predict immunotherapeutic responses and/or strategies that improve response to ICB (142). Importantly, PD-L1 IHC was performed similarly on FFPE archival (KEYNOTE-012) or excisional biopsy specimens (KEYNOTE-028) with a central laboratory that used the 22C3 anti-human PD-L1 antibody (Merck & Co.) PD-L1 expression was determined by combined positive score (CPS) defined as the number of PD-L1+ cells (tumor cells, lymphocytes, and macrophages) divided by the total number of tumor cells, multiplied by 100. According to that assay, a tumor is considered to have positive PD-L1 expression when CPS is greater than or equal to 1.

In the phase 1b JAVELIN trial, 168 heavily pretreated patients with metastatic breast cancer, regardless of subtype or PD-L1 status, were treated with the PD-L1 inhibitor, avelumab (143). Of the 168 patients, 72 had HR+/HER2- disease and the ORR for this group was 2.8% (2/72) compared to 5.2% (3/58) in the TNBC group. The median duration of response was not reached. In addition, subgroup analysis by PD-L1 status did not reveal any trend in efficacy. Given the low ORR, avelumab was determined to have limited therapeutic benefit as monotherapy in patients with metastatic HR+/HER2- breast cancer. Altogether, the KEYNOTE-028 and JAVELIN trials revealed the limited single-agent efficacy of ICB in HR+ breast cancer, particularly in heavily pretreated disease. The limited response to ICB monotherapy led to the inclusion of chemotherapy and other systemic therapeutics that may have synergism with ICB, a strategy used in TNBC.

### ICB in Combination With Chemotherapy for HR+ Breast Cancer

Although chemotherapy has historically been considered immunosuppressive (144), robust preclinical and clinical data show that cytotoxic drugs enhance tumor immunity and have synergism with ICB. It is thought that after exposure to chemotherapy, release of tumor cell neoantigens from dying cancer cells can activate an anti-tumor immune response by inducing CD8+ T cell infiltration and activation. Those findings are important because, as discussed earlier, TILs are an independent predictor of response to chemotherapy (40). Pre-clinical models have shown that the tubulin-targeting drug, paclitaxel, increases tumor cell permeability to granzyme-B (released from CTLs) (145) and upregulates major histocompatibility complex (MHC) class I expression on cancer cell lines (146) to induce tumor cell immunogenicity. Importantly, in the phase III IMpassion130 trial, which tested adding atezolizumab (anti-PD-L1) to nab-paclitaxel (albumin-bound paclitaxel) demonstrated a significant improvement in PFS and a clinically meaningful improvement in OS in first-line treatment of PD-L1+ metastatic TNBC (16). Those results led to the FDA approval of atezolizumab in combination with nab-paclitaxel in PD-L1+ (SP142 IC≥1) metastatic TNBC, establishing the first ICB approval in breast cancer. More recently, pembrolizumab, in combination with different chemotherapy agents, was also approved for the treatment of locally advanced or metastatic TNBC, based on results from the KEYNOTE-355 trial (14). ICB in combination with nab-paclitaxel or chemotherapy is only approved for PD-L1-positive locally recurrent/advanced or metastatic TNBC, and while there are responses, the majority of patients eventually experience disease progression (147, 148).

Given the promising results using chemotherapy with ICB in TNBC, there has been an effort to replicate similar strategies in HR+ breast cancer. Like the early monotherapy trials, the initial chemotherapy plus ICB combination trials focused on heavily pretreated patients in the metastatic setting. The first of these trials used eribulin as a combination agent. Eribulin is a microtubule inhibitor that, in addition to antimitotic activity, has been shown to reverse epithelial-mesenchymal-transition (EMT) (149) and decreased numbers of FOXP3 and PD-L1 expression as measured through IHC (150). In the phase II trial, eribulin (E) with or without pembrolizumab (P) was evaluated in 88 (44 E+P, 44 E) patients with HR+/HER2- metastatic breast cancer (151). In this cohort, the patients had received at least two prior lines of endocrine therapy and up to two lines of chemotherapy. The addition of pembrolizumab to eribulin did not add any benefit to median PFS (4.1 *vs* 4.2 months, p=0.38). In addition, PD-L1 status, TILs and TMB were not associated with median PFS. Importantly, 54.6% of patients who received E+P experienced grade 3-4 adverse events, including 2 treatment related deaths.

Another trial tested the combination of capecitabine with pembrolizumab (152). Capecitabine is a prodrug of 5-Fluorouricil (5-FU), which inhibits DNA replication. The ability of 5-FU to enhance immune activity is debated. In preclinical studies, 5-FU has been shown to increase expression of carcinoembryonic antigen (CEA) in breast cancer cell lines (153) and reduce the number of myeloid derived suppressor cells (MDSCs) in murine models (154). However, in patients with pancreatic cancer, 5-FU failed to elicit a decrease in MDSCs (155) or a decrease in MDSC promoting cytokines (156). In this phase II trial, 30 patients with metastatic breast cancer and previous endocrine resistance (14 with HR+ disease and 16 with TNBC) were treated with a combination of pembrolizumab and capecitabine (152). Among the 29 evaluable patients, the median PFS was 4 months, the ORR was 14% and the clinical benefit rate (CBR) was 28%. The response rates did not differ between subtypes. Given this relatively modest response rate, this regimen was deemed not worthy of further study in breast cancer.

The lack of clinical benefit in both the eribulin and capecitabine combination trials may indicate that these chemotherapeutic agents do not sufficiently increase tumor immunogenicity to a level that enhances ICB efficacy. Targeted chemotherapy in the form of antibody drug conjugates (ADC) may better augment tumor immunogenicity, as suggested by the efficacy of the anti-Trop-2-SN-38 ADC sacituzumab govitecan in heavily pretreated HR+ metastatic breast cancer refractory to endocrine therapy (157). To test whether ADC therapy synergizes with ICB, the ongoing SACI-IO HR+ trial is investigating whether pembrolizumab added to sacituzumab govitecan improves progression-free survival compared to sacituzumab govitecan alone in PD-L1+ metastatic HR+ disease (NCT04448886). However, an alternate explanation for the lack of efficacy may be the fact that these trials evaluated ICB in heavily pretreated patients with metastatic breast cancer. Compared with metastatic tumors, primary breast cancers have more TILs and higher PD-L1 expression (60, 158), both of which are predictive of response to immunotherapy (2, 25, 136), leading to the hypothesis that ICB could have a more impactful role in the neoadjuvant setting.

In the ISPY-2 trial, 40 HR+/HER2- and 29 TNBC patients were treated in the neoadjuvant setting with pembrolizumab in combination with standard chemotherapy (paclitaxel followed by doxorubicin and cyclophosphamide) (13). pCR was used as the primary endpoint and the study aimed to determine if the combination of pembrolizumab with neoadjuvant chemotherapy was likely to succeed in the phase III clinical trial. In the HR+ subgroup, the addition of pembrolizumab yielded a higher rate of pCR compared to that of the chemotherapy arm (34% *vs* 13%, respectively). Benefit was also seen in the TNBC cohort (60% *vs* 20%). Importantly, the ISPY-2 trial concluded that the predictive probability of this treatment strategy succeeding in a phase III, HR+/HER2- trial was 99.6%. Pembrolizumab was the first agent of ten studied to graduate in the HR+/HER2- subtype in the ISPY-2 trial and may suggest that further stratification or targeting of HR+ patients would reveal which populations would benefit from ICB. With these promising results, the idea of successful implementation of ICB in the “immunologically cold” HR+ subtype was revitalized. Specifically, this arm of the ISPY-2 trial showed that by focusing on patients in the early setting, ICB may have a beneficial role in HR+ disease. Moreover, the results suggest that there may be informed ways to identify the right chemotherapy combinations, particularly for breast cancers that are not innately sensitive to ICB. However, further analyses of long-term outcomes are needed to critically evaluate if the combination of ICB and chemotherapy will provide long-term benefit compared to the potentially life-threatening adverse effects that may be associated with such combinations. Importantly there are two phase III clinical trials evaluating ICB in HR+/HER2- breast cancer in the preoperative setting. In the first, the activity of pembrolizumab in combination with standard chemotherapy and hormone therapy in the preoperative and adjuvant setting versus chemotherapy and hormone therapy alone is being evaluated in stage I-III HR+ breast cancer patients (NCT03725059). Another phase III trial is evaluating the safety and efficacy of adding nivolumab (anti-PD-1) in the preoperative and adjuvant setting in combination with standard therapy in stage II/III HR+/HER2- breast cancer patients (NCT04109066). Early use of ICB in HR+ breast cancer may provide insight into how ICB fits into the clinical care of HR+ breast cancer patients.

### ICB in Combination With Other Treatment Modalities for HR+ Breast Cancer

ICB in combination with chemotherapy for the treatment of HR+ breast cancer has shown some success, particularly in the neoadjuvant setting; however, it remains unclear if chemotherapy is sufficient to reverse these immunologically cold tumors. Importantly, there is a wide variety of treatment options for patients with HR+ breast cancer including targeted molecules and radiation. Thus, there has been an interest in the synergistic potential of these other treatment modalities.

PARP inhibitors olaparib (159) and talazoparib (160) are approved for the treatment of advanced breast cancers with BRCA1/2 germline mutations. These drugs block the base excision repair pathway, leading to DNA damage, and induce synthetic lethality in BRCA mutant breast cancers (161). More recently, PARP inhibitors were shown to generate an antitumor response through activation of the STING (stimulator of interferon genes) pathway (162). In murine models, STING-dependent infiltration of CD8+ T cells was demonstrated to be required for response to olaparib (163). Furthermore, PARP inhibitors were found to increase PD-L1 expression in breast cancer cell lines and murine models (164). Thus, PARP inhibitors represent a promising combination therapy with ICB. In the phase II MEDIOLA trial, the efficacy of durvalumab (anti-PD-L1) in combination with olaparib was assessed in 34 patients with metastatic breast cancer (13 had HR+ disease, 21 had triple negative disease) with germline *BRCA*1/2 mutations (165). Of the 30 evaluable patients, this combination strategy achieved (at 12 weeks) was a disease control rate (DCR) of 85% with a median PFS of 8.2 months. Median OS was comparable between the subtypes (HR+ = 22.4; TNBC = 20.5) and was comparable to either agent used as monotherapy. Interestingly, the efficacy was dependent on the extent of prior treatment. Patients with 0-1 prior lines of chemotherapy experienced a longer median duration of response (12.9 months *vs*. 5.5 months) and a longer median PFS (11.7 months *vs* 6.5 months) compared to patients with 2 prior lines of chemotherapy. With the exciting results from the MEDIOLA trial, PARP inhibitors gained much interest as a combination strategy with immunotherapy. In a second arm of the ISPY-2 trial, neoadjuvant durvalumab and olaparib in combination with paclitaxel (DOP) were compared to paclitaxel alone in patients with high risk, HER2- breast cancer (52 HR+ and 21 TNBC), regardless of BRCA status (166). Both subtypes yielded a significant clinical benefit with an estimated pCR of 28% in HR+ patients and 47% in TNBC patients. The estimated probability of success in a phase III clinical trial for DOP in HR+ patients was 74.5%. Importantly, this trial showed PARP inhibitors have synergism with checkpoint blockade, regardless of BCRA status. However new data indicate that PARP inhibitors may negatively modulate the TME by inducing suppressive TAMs and therefore should be further evaluated (167).

CDK4/6 inhibitors (abemaciclib, palbociclib, ribociclib) inhibit cell cycle progression and are approved for patients with HR+/HER2- metastatic breast cancer (168, 169). In addition to cell cycle inhibition, abemaciclib has been shown to enhance immunogenicity within the TME through increased antigen presentation, increased CD8+ T cell infiltration, and decreased T-reg infiltration and proliferation (170) and PD-L1 expression (171). Goel et al. first reported that abemaciclib plus anti-PD-L1 induced durable responses in preclinical models of HR+ breast cancer and mice deemed tumor free were protected from subsequent tumors when re-challenged with tumors, suggesting sustained immune memory (170). Similarly, Schaer and colleagues showed synergism between abemaciclib and PD-L1 inhibitors in murine models (172). These results were confirmed in the NeoPalAna trial, in which patients with primary HR+ breast cancer underwent tumor biopsies prior to palbociclib and then at 2 and 12 weeks of treatment. Gene expression profiling revealed that the addition of palbociclib to endocrine therapy enhanced anti-tumor immunity, as seen in the mouse models (170). Thus, CDK4/6 inhibition is a potential candidate to combine with ICB in patients with HR+ breast cancer.

In cohort C of the phase 1b JPCE trial, the efficacy of abemaciclib in combination with pembrolizumab was assessed in 28 patients with endocrine resistant, metastatic HR+/HER2- disease (173). The inclusion criteria were 1-2 prior treatments with chemotherapy, no previous CDK4/6 or ICB treatments and ECOG PS ≤1. At 24 weeks, 8 patients achieved a confirmed partial response (ORR 28%). The DCR was 82%, median PFS was 8.9 months and OS was 26.3 months. We compared to abemaciclib monotherapy in a similar patient population (MONARCH1) (174), the clinical benefit was not only numerically but also statistically significantly improved. Combination therapy resulted in numerically higher rates of elevated transaminases; however, the overall safety profile was considered generally tolerable. Importantly, in another cohort (cohort D) of the JPCE trial, the safety of abemaciclib in combination with pembrolizumab and the aromatase inhibitor (AI) anastrozole was assessed in 26 patients with locally advanced or metastatic HR+/HER2- breast cancer (175). Preliminary safety results revealed a high level of grade 3/4 AEs including 8 patients with neutropenia, 6 patients with elevated alanine aminotransferase and 2 therapy-related fatalities (pneumonitis). Given the high level of adverse events in cohort D, further development of this triple approach has been discontinued (176).

Radiotherapy is a well-established local therapy that has shown a survival benefit in high risk and early-stage breast cancer (177). Historically, the benefits of radiotherapy were attributed to cell-autonomous death from overwhelming DNA damage. However, further analysis revealed radiation-induced DNA damage can stimulate a systemic immune-mediated anti-tumor response, known as the abscopal effect (178). Importantly, a recent trial in TNBC found the combination of pembrolizumab and radiotherapy resulted in partial and durable responses in 33% of patients (3 of 9) (179). Thus, synergistic effects of radiation with immunotherapy were tested in HR+ breast cancer patients. In a phase II trial, the efficacy of pembrolizumab in combination with palliative radiotherapy was assessed in 8 patients with HR+ metastatic breast cancer (180). There were no objective responses observed among 8 patients, resulting in early closure of the study. In contrast, a similar trial in patients with TNBC demonstrated a partial response in 33% and stable disease in 11% of the 17 patients (181). While the combination of radiotherapy plus pembrolizumab produced no objective responses in the HR+ patient population, it is important to note that the patients in this study were very heavily pretreated, and the number of patients was small, making it difficult to draw definitive conclusions from this trial. Determining the potential benefits of combining systemic ICB treatment with local radiotherapy likely warrants future studies. As with other combination strategies, finding the optimal patient population and sequence of treatment may yield clinical benefit.

### Other Checkpoint Inhibitors in HR+ Breast Cancer

Other T cell checkpoints other than CTLA4 and PD-1 have been identified and have been targeted for anti-cancer therapy and has been previously reviewed (182). For example, T cell immunoreceptor with immunoglobulin and ITIM domain (TIGIT) is upregulated by immune cells, including activated T cells, natural killer cells, and regulatory T cells and T-cell immunoglobulin and mucin domain 3 (Tim-3) is a checkpoint receptor expressed by a wide variety of immune cells as well as leukemic stem cells. Both TIGIT and Tim-3 are promising new target for cancer immunotherapy (183, 184). There are currently Phase I trials evaluating TIGIT and Tim-3 including for patients with breast cancer. Future work will determine if these other checkpoints will be relevant for HR+ breast cancer. However, given the low recruitment of both T cells and NK cells as well as low tumor and immune cell expression of PD-L1 in HR+ breast cancer, it will be important to identify other ways to modulate the TME to successfully activate an anti-tumor immune response in HR+ breast cancer. In a study of approximately 450 HR+ tumors treated with AI, AI-resistant luminal B tumors revealed an upregulation of immune checkpoint components, particularly indoleamine 2, 3-dioxygenase 1 (IDO1), lymphocyte-activation gene 3 (LAG3), and PD-1, which are associated with negative regulation of T cell activation and function (185, 186). Additionally, downregulation of the human mutL homolog 1 (MLH1), which is vital in mismatch DNA repair, was also identified in AI-resistant tumors. IDO1 expression in intraepithelial myeloid cells was strongly associated with PD-L1 expression on carcinoma cells and PD-1 and LAG3 expression on TILs. This study also provided evidence that IDO1+ macrophages correlated with CD8+ T cells and might suggest a mechanism of T cell suppression (187). The IDO1 inhibitor, epacadostat, has been recently tested in clinical trials and has shown both safety and activity, especially in combination with Ipilimumab (anti-CTLA4) in metastatic melanoma (188–190). Taken together, these findings suggest that subsets of HR+ breast cancer may benefit from IDO-targeted treatment and may warrant further study.

There is evidence that estrogens can modulate PD-1/PD-L1 expression in endometrial tissue (191) and on immune cells (192, 193), and PD-L1 expression on HR+ breast cancer cells *in vitro* (194), which may limit the function of T cells in HR+ breast cancer. Anti-estrogen therapy has been shown to amplify immunotherapeutic target expression of α-lactalbumin on breast cancer cells. α-lactalbumin is a lactation protein negatively regulated by estradiol-17β and has been a target of vaccination in TNBC (195). Therefore, anti-estrogen therapy may downregulate PD-L1 expression and increase other targets, acting as a priming event for concurrent therapy to induce an anti-tumor immune response (196, 197). In addition, in preclinical studies, steroid-like selective ER degrader (SERD) fostered immune stimulatory activity by inhibiting suppressive myeloid cells and, in combination with anti-PD-L1 therapy, induced tumor regression and activation of anti-tumor macrophages and T cells (197).

Anti-estrogen therapy has also been shown to regulate CD47 expression. CD47 is a widely expressed cell-surface receptor that inhibits phagocytosis signaling through its engagement with SIRP1α on macrophages. High expression of CD47 correlates with worse survival in both HR+ and HER2+ breast cancer but not TNBC (198). CD47 is highly expressed in endocrine therapy-resistant tumors, suggesting a new role for CD47 in mediating anti-estrogen resistance (199). Targeting the unfolded protein response, GRP78, re-sensitized tumors to anti-estrogen treatment and correlated with increased levels of calreticulin and high molecular group box 1 (HMGB1) protein, indicating activation of immunogenic cell death pathways (200). Co-expression of GRP78 and CD47 is associated with a significant decrease in survival in HR+/HER2- breast cancer (200). In addition, CD47 has been shown to have increased expression on HR+ breast cancer cells following hypoxia (201). Furthermore, H3K27ac ChIP-Seq profiling revealed downstream super enhancers associated with CD47 in an HR+ breast tumor and HR+ cell lines but not TNBC tumors or cell lines (202). Anti-CD47 therapy has been extensively studied for the treatment of other cancers to eliminate tumor cells through macrophage phagocytosis (203, 204). Such strategies may offer therapeutic utility in the treatment of HR+ breast cancers, especially those resistant to endocrine therapy (119–124). Given that HR+/HER2- tumors generally do not present with the T cell inflamed phenotype, developing alternative strategies for activating anti-tumor immune responses remains an unmet need. In that regard, use of current as well as novel technologies should be employed for deep characterization of HR+ breast tumors with the goal of elucidating immune mechanisms in the TME that can incite the next generation of clinical trials to enhance immune signaling in HR+ disease.

## Methods for Interrogating the TME to Reveal Novel ICB targets

Recent advances in molecular and genomic profiling, as well as multi-plex tissue analysis have allowed a deep understanding of the TME and have revealed novel mechanisms and opportunities to overcome immune suppression in HR+ breast cancer, as reviewed here. Further strategies aimed at more deeply characterizing the TME of HR+ breast cancer and contrasting it to immune rich, ICB-responsive tumors may greatly facilitate development of novel strategies for the use of ICB in HR+ breast cancer. In this section we aim to review current technologies used to explore the TME and include both advantages and disadvantages to each strategy.

### Immunohistochemistry

Several studies have demonstrated that high TILs and PD-L1 expression have been linked to predictive benefit of anti-PD-1/L1 therapy in TNBC (16). Importantly, these are assays that require formalin-fixed paraffin-embedded (FFPE) tissue. Significant heterogeneity of PD-L1 protein expression identified by IHC has been reported in several studies (25, 139). This observed heterogeneity could be caused by the wide array of IHC platforms and antibodies, as well as pathological scoring methods and cutoffs. In addition, the use of tissue microarrays (TMAs) may limit conclusions. A recent study revealed that out of 118 tumors used to compare TMA with whole slide observations, 49% of the TMA tumor results were false negatives, whereas whole tissue sections that the TMAs were derived from revealed positive staining (25). In addition, TILs and/or PD-L1 may not hold the best predictive or prognostic value in HR+ breast cancer.

Macrophages comprise a significant portion of the breast TME (205) and have recently been the focus of several studies using IHC to interrogate HR+ breast tumor samples (206–211). To detect macrophages, IHC studies have most commonly used antibodies against CD68 (207–211) and CD163 (206). Notably, Luminal A (LumA) tumors have been shown to have fewer macrophages compared to Luminal B (LumB) tumors (206, 207). The increased numbers of macrophages in LumB tumors have been associated with an increase of Ki67+ proliferative tumor cells (206, 207), high tumor grade (206, 207, 210, 211) and loss of ER (206–210). In addition, tamoxifen-resistant patients have been shown to have increased numbers of CD163+ macrophages in the TME compared to tamoxifen sensitive patients (212). Increased density of macrophages in breast tumors has been suggested to predict poor prognosis (207), although some studies have been unable to confirm this association (209). Tumor cells can evade macrophage phagocytosis by overexpressing the ‘don’t eat me’ signal CD47, inducing immune escape (213). Yuan and colleagues focused on capturing the interaction between CD68+ macrophages and CD47+ tumor cells in 217 primary breast tumor samples (n=96 HR+) (210). CD68+ macrophages were frequently seen within close proximity of CD47+ tumor cells in all breast cancer subtypes. Nearly 40% of HR+ tumors were characterized with high expression density of both CD47 and CD68, which implies potential crosstalk between tumor cells and macrophages, and the formation of an immunosuppressive TME, at least in a subset of HR+ breast tumors. The combined high expression of CD47 and CD68 was associated with poor prognosis in patients with HR- breast tumors, but no association was observed in patients with HR+ tumors (210).

### Genomic and Transcriptomic Profiling

Genomic and transcriptomic profiling of bulk tumor tissue has vastly expanded our knowledge of immune cell phenotypes in HR+ breast tumors. Recently, an extensive immunogenomic profiling of cancers analyzed by The Cancer Genome Atlas (TCGA) were characterized for assessment of total lymphocyte infiltrate, immune cell fractions, gene expression, neoantigen prediction as well as T cell receptor and B cell receptor (214). The analysis included 508 LumA and 191 LumB tumors and revealed six clusters of immune subtypes. The study revealed that a majority (86%) of LumA tumors belonged to either the wound-healing, interferon gamma IFN*γ* dominant or inflammatory immune subtype (214). In contrast, 95% of LumB tumors belonged to either the wound-healing, IFN*γ* dominant or lymphocyte-depleted subtype. The wound-healing subtype was characterized by an increased expression of angiogenic genes, a high proliferation rate and a trend toward T helper 2 (Th2) dominant lymphoid infiltrate. The IFN*γ* dominant subtypes had increased signatures of CD8^+^ T cells and a substantial number of lymphocytes compared to macrophages. In contrast, the inflammatory subtype was characterized with increased levels of Th17 gene signatures and a balanced macrophage/lymphocyte ratio. Lymphocyte-depleted subtypes had elevated levels of macrophage signatures, notably M2, with Th1 suppressed response (214). This study challenges the previous paradigm of immunologically cold HR+ breast tumors and highlights the importance of various immunosuppressive mechanisms that are active within HR+ breast tumors.

CIBERSORT (215) is a computational method that quantifies the proportion of 22 functional immune subsets within bulk tissue gene expression profiles. Ali and colleagues used CIBERSORT to analyze bulk gene expression profiles of 10,988 breast tumors (n=5,807/53% HR+/HER2-) from 56 publicly available datasets (117). Specifically, this study aimed to determine the relationship between TME composition and molecular subtype, survival and response to chemotherapy. In HR+ tumors, the presence of M0 macrophages and regulatory T cells were associated with poor prognosis (117), which was later confirmed by another group studying the prognostic significance of tumor-infiltrating immune cells in breast cancer (216). Notably, the HR+ tumors lacking immune infiltration were associated with intermediate or similar survival outcomes compared to HR+ tumors with high or low immune infiltrates. Thus, in this large cohort, the presence of immune cells was not prognostic of outcome in HR+ breast tumors (117).

Recent work from Cassetta and colleagues has identified a TAM signature that is highly enriched in aggressive breast cancer subtypes and associated with shorter disease-specific survival, interestingly the signature was found in all subtypes, providing evidence of heterogeneity in each subtype (217). Bense et al. characterized the immune cell composition and functionality of 7,270 breast tumors (n=4,094 HR+/HER2-) (118). This study used raw microarray expression data from primary breast tumors that were publicly available in the Gene Expression Omnibus (GEO) database (218). CIBERSORT was used to estimate immune cell type fractions, and the relationship between the immune cell type fractions and five different immune signatures was determined (37, 219–222). In the HR+/HER2- cohort, a higher fraction of M1 macrophages was predictive of pCR to neoadjuvant chemotherapy and prognostic of DFS and OS. A high CD4+ follicular helper T cell signature score was associated with prolonged DFS and OS (37). Additionally, a high CD8+ T cell exhaustion signature score was associated with shorter DFS in patients with HR+ tumors regardless of HER2 status, suggesting the hypothesis that CD8+ T cell exhaustion could be related to immune evasion in HR+ breast cancer. However, this observation was not confirmed in the subgroup analyses focusing only on HR+/HER2- or HR+/HER2+ tumors.

In another effort to study the complex relationship between ER positivity and inflammatory response, gene expression of 195 breast tumors was compared to matched adjacent normal tissue (223). Surprisingly, HR+ tumors had a decrease in macrophage related gene signatures compared to adjacent normal tissue samples. In addition, there was an inverse correlation between the tumor estrogen pathway expression and the tumor macrophage score, suggesting that high levels of estrogen signaling have suppressive effects on macrophages in the breast tumor microenvironment.

### Single-cell Analysis

Single-cell RNA sequencing allows precise cell state mapping and reveals individual immune cell phenotypes within tumors. One of the early efforts to characterize the immune landscape of breast tumors with single-cell RNA sequencing was made by Chung et al., who analyzed a total of 175 immune cells from 11 breast cancer patients (18). The detected TAM populations were enriched for genes related to immunosuppression and promotion of tumorigenesis. Azizi et al. performed more extensive profiling of the breast TME (n=8 primary breast tumors; 5/8 HR+) (224). They observed an increased diversity of immune cell states in breast tumors compared to normal breast tissue. Notably, when focusing on the macrophage populations in these breast tumors, both immunosuppressive and immunostimulatory related gene signatures were frequently expressed in the same cells. The positive correlation of both pro- and anti-tumor associated genes challenges the previously suggested and mutually exclusive M1 and M2 activation states and highlights the continuous spectrum of activation states of TAMs in breast cancer.

Molecular profiling with mass cytometry (CyTOF) of 138 breast cancer patients (39% LumA, 51% LumB) using 34 immune cell targets and 38 tumor-centric antibodies with mass cytometry (225) revealed epithelial, endothelial, fibroblasts and immune cells. The most abundant immune cell types were T cells and myeloid cells. Twenty unique CD4+ and CD8+ T cell clusters were identified. A minor proportion of both LumA and LumB tumors harbored PD-1+ T cells. However, the PD-1+ T cells were more abundant in LumB tumors compared to LumA tumors. When focusing on the co-expression of PD-1, CTLA-4 and activation marker CD38 across the various T cell phenotypes, the authors found PD-1^int^CTLA-4^-^CD38^-^ T cells were more frequent in LumA tumors compared to LumB tumors. Notably, a minor subset of all HR+ tumors had increased frequencies of PD-1^high^CTLA-4^+^CD38^+^ T cells and T regs, suggesting that a specific subset of HR+ breast cancer patients could be candidates and benefit from immune checkpoint blockade therapies. In addition to various T cell phenotypes, 19 unique myeloid cell clusters were identified, which were further divided into five categories: 1) CD14-expressing monocytes (CD14^+/int^CD16^-/+^), 2) early immigrant macrophages (HLA-DR^int^CD192^+^), 3) tissue-resident macrophages (CD206^+^HLA-DR^int^), 4) TAMs (CD64^high^HLA-DR^high^) and 5) myeloid-derived suppressor cells (HLA-DR^-/low^). The composition of these heterogenous myeloid cell categories varied according to the tumor grade and histopathological subtype. When focusing on PD-L1 expression in the myeloid compartment, PD-L1^+^ TAMs were more abundant in LumB compared with LumA tumors. The frequency of PD-L1^+^ TAMs was also higher in grade 3 than in grade 2 tumors (225).

### Multiplex Tissue Analysis

Although the previously presented studies using CyTOF, bulk RNA and single-cell RNA sequencing provide comprehensive insight on the heterogeneity of cell phenotypes and states across breast cancer subtypes, these methods lack the spatial information of the tissue architecture and do not provide an opportunity to evaluate the relationships of single cells in the spatial context. Several single-cell imaging techniques have been used to address this challenge, including multiplex IHC (226), cyclic immunofluorescence (CyCIF) (227), CODEX (228), multiplexed ion beam imaging (MIBI) (229) and imaging mass cytometry (IMC) (230–233). However, to date, only two publications (234, 235) have focused on HR+ breast tumors with the previously mentioned single-cell pathology techniques.

Jackson et al. studied the complex single-cell phenotypes and their spatial location in breast tumors with IMC (235). The aim was to quantify spatial inter- and intratumor heterogeneity of the breast TME on a single-cell level. In this study, tissue microarrays (TMAs) composed of 352 breast tumors (n=175 HR+/HER2-) were analyzed. Diverse cell phenotypes of endothelial, immune, stromal, and tumor cells were identified using 35 antibodies. Populations of fibroblasts, endothelial, and immune cells were present at similar densities in each breast tumor subtype. When looking at the cell-cell interactions, a subset of microenvironment communities was enriched for only T cells, while communities consisting of large networks of T and B cells across the samples were also identified, possibly implying the existence of TLS. The microenvironment communities that were enriched in fibroblasts had decreased numbers of immune cells, which supports the hypothesis of fibroblasts as mediators of immune exclusion (236). Interestingly, HR+ tumors harbored a range of fibroblast-enriched stromal environments, and only a subset of HR+ tumors contained rare and localized immune-enriched stromal environments (236).

As a follow-up study, the effect of somatic alterations on the cellular composition of breast tumors and the architecture of the tumor microenvironment was studied by coupling single-cell IMC data to the multiplatform genomic profiling with transcriptomic, Copy Number Aberration (CNA) and microRNA data (234). A total of 483 primary breast tumor samples (30.8% LumA, 21.1% LumB) from the Molecular Taxonomy of Breast Cancer International Consortium (METABRIC) cohort collected between 1985 and 2005 were included in this comprehensive phenogenomic analysis. IMC analysis revealed various epithelial, stromal and immune cell phenotypes. Breast cancer subtypes were determined with PAM50 gene expression profiles. Within this cohort, the only immune cell phenotype enriched in the HR+ tumors were Vim^+^Slug^-^ macrophages, which were enriched in the LumB subtype. LumA tumors were characterized by enrichment of several distinct fibroblast and myofibroblast phenotypes, that were not found as extensively within the other genomic breast cancer subtypes. The expression of hormone receptors and various cytokeratins within epithelial cells also differed between LumA and B tumors. Beyond cell phenotyping, the authors showed how certain epithelial, stromal, and immune cell phenotypes were linked with underlying driver gene alterations and CNAs. The number of proliferative cells, macrophages and T cells increased with genomic instability. The authors concluded that the cell phenotypes are diverse across the breast cancer genomic subtypes and that the luminal tumors were composed of a mixture of cell phenotypes rather than of a single dominant cell population. The authors noted that the phenotypic compositions of luminal tumors seemed to be largely affected by both somatic alterations and the transcriptional programs induced by ER signaling, which is consistent with previous studies suggesting that endocrine therapy expands the phenotypic clones that are under-presented at the time of diagnosis (237).

**Figure 1:**
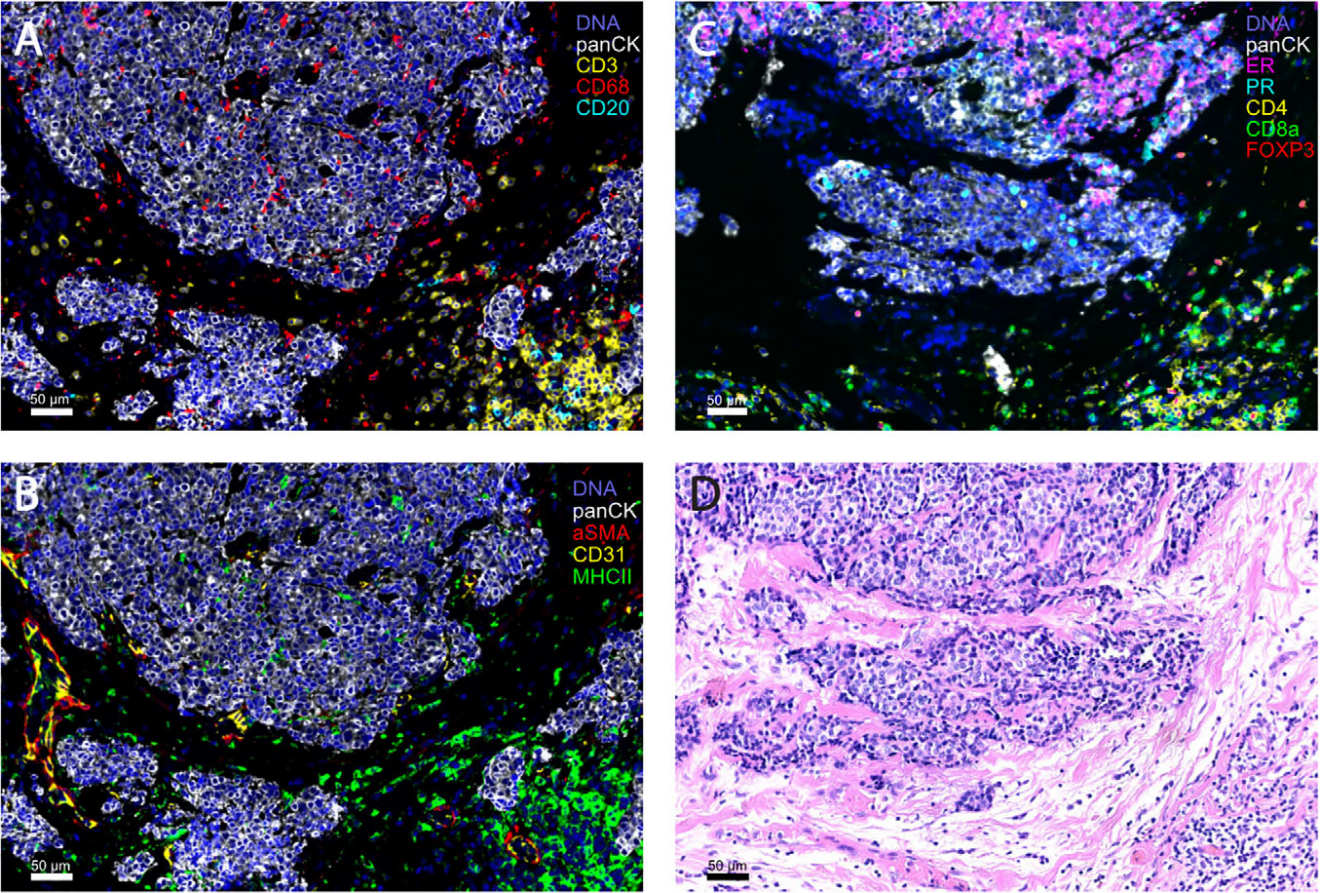
Representative images of HR+ breast tumors obtained with highly multiplex cyclic immunofluorescence (CyCIF) imaging **(A-C)** and the corresponding H&E section **(D)**. CyCIF is a robust tool for the investigation of the complexity of the tumor microenvironment, by linking the cell type with spatial information. **(A, B)** are from the same formalin-fixed, paraffin embedded (FFPE) slide and **(C, D)** are both from serial sections of a primary breast tumor (invasive ductal carcinoma, HR+HER2-).

The field of single-cell analysis is constantly growing, and these previously mentioned modern techniques (226–230) will greatly contribute to our understanding of the complexity of HR+ breast TME. The evaluation of large tumor areas with high-throughput, whole tissue section imaging methods, such as CyCIF (227) (**Figure 1**), and in the future, 3D modeling of tumor architecture will provide a deeper knowledge of potential novel biomarkers and therapeutic targets in HR+ breast cancer.

## Discussion

In this review, we explored the immune microenvironment of HR+ tumors, along with pre-clinical approaches and clinical investigations in HR+ breast immuno-oncology. We shed light that in fact, HR+ tumors are not devoid of immune infiltration. Next generation sequencing and various histologic approaches show that there is an endogenous, albeit limited, immune response to HR+. However, an immunosuppressive TME characterized by TAMs and low levels of tumor HLA-I expression, limits anti-tumor immune activity and may be the culprit for T cell and NK cell exclusion. Additionally, low PD-L1 expression on HR+ tumors and infiltrating immune cells may further limit the efficacy of PD-1/PD-L1 targeted therapy. We further posit that deep mechanistic and functional characterization of the immunologic aspects of the TME in HR+ breast cancer is urgently needed. Comprehensive profiling of HR+ tumors at baseline and on treatment, combined with pre-clinical study, should lead to improved understanding of the TME and reveal mechanisms by which HR+ breast cancers obstruct T cell and NK cell infiltration, evoke low levels of HLA class I expression and are broadly resistant to ICB.

Patients with metastatic HR+ breast cancer have shown limited response to checkpoint inhibition, and clinical investigations into this patient population has thus been limited. Importantly, the ISPY-2 trial (neoadjuvant chemotherapy plus pembrolizumab) revealed, for the first time, a clinically effective immunotherapeutic strategy for patients with HR+ breast cancer. Furthermore, data from PARP and CDK4/6 inhibitor combinations with checkpoint inhibitors are promising. There are currently multiple on-going clinical trials assessing the combination of checkpoint blockade with PARP inhibitors (NCT03594396, NCT02849496) and CDK4/6 inhibitors (NCT02778685, NCT02779751, NCT0314728, NCT03147287, NCT03573648, NCT03294694) in HR+ breast cancer. Identifying the appropriate combination strategy, sequencing of treatment and patient population is critical to the optimal use of ICB in HR+ breast cancer. It is possible that targeting alternative checkpoints such as TIGIT and Tim-3, largely expressed on T cells, as well as therapies against NK cell checkpoints, such as killer cell immunoglobulin-like receptors (KIR; also known as CD158) and NKG2A, will be effective in HR+ breast cancer. However, effective targeting of such checkpoints will likely require appropriate recruitment strategies aimed at getting T cells and NK cells into the tumor.

We present novel immunotherapy strategies that warrant new lines of investigation, such as adding other agents (i.e. targeted therapies such as small molecule drugs or monoclonal antibodies) that impact the TME, thereby increasing TIL (both T cell and NK cell) infiltration and enhancing response to ICB. As an example, it has been shown that CDK4/6 inhibitors result in tumor-expression of cytokines that promote T cell recruitment. We also highlight the role of immune cells other than T cells, such as TAMs, which are abundant in HR+ breast tumors and play an immunosuppressive role in the TME. Further work is needed to better characterize TAMs in HR+ breast cancer, which will inform how to move forward in targeting these cells for anti-cancer therapy. Taken together, these findings will be critical for next generation clinical trials to harness the power of immunotherapy in HR+ breast cancer.

## Author Contributions

All authors contributed to the article and approved the submitted version.

## Funding

EM acknowledges the Rob and Karen Hale Distinguished Chair in Surgical Oncology for support. JG is supported by the Dana-Farber/Harvard Cancer Center (DF/HCC) Specialized Program of Research Excellence (SPORE) in Breast Cancer P50 CA1685404 Career Enhancement Award, The Susan G. Komen Foundation Career Catalyst Award CCR18547597, The Terri Brodeur Breast Cancer Foundation, The Saverin Family Foundation at Dana-Farber Cancer Institute and The Ludwig Center at Harvard. SM is supported by a Department of Defense BCRP Era of Hope Expansion Award W81XWH2010472 and a METAvivor Translational Research Award, Chicago Metsquerade Presented in Memory of Lauren Smoke. TV acknowledges grant support from the Finnish Medical Foundation, Relander Foundation, Turku University Foundation, Maud Kuistila Memorial Foundation, the Finnish Society of Oncology, and the Cancer Society of Southwest Finland.

## Conflict of Interest

EM has been compensated for participation on Scientific Advisory Boards for Astra-Zeneca/Medimmune, Celgene, Genentech, Exact Sciences (formerly Genomic Health), Merck, Peregrine Pharmaceuticals, SELLAS Lifescience, and Tapimmune and has had clinical trial support to her former institution (M.D. Anderson Cancer Center) from Astra-Zeneca/Medimmune, EMD-Serono, Galena Biopharma and Genentech, and current institution from Genentech *via* a SU2C grant. EM has also had sponsored Research Support to the laboratory from GSK and Eli Lilly.

JG is a consultant for Glaxo-Smith Kline (GSK), Codagenix, Verseau, Kymera and Array BioPharma and receives sponsored research support from GSK, Array BioPharma and Eli Lilly.

ST receives institutional research funding from AstraZeneca, Lilly, Merck, Nektar, Novartis, Pfizer, Genentech/Roche, Immunomedics, Gilead Exelixis, Bristol-Myers Squibb, Eisai, Nanostring, Cyclacel, Odonate, and Seagen; has served as an advisor/consultant to AstraZeneca, Lilly, Merck, Nektar, Novartis, Pfizer, Genentech/Roche, Immunomedics, Bristol-Myers Squibb, Eisai, Nanostring, Seagen, Puma, Sanofi, Celldex, Paxman, Puma, Silverback Therapeutics, G1 Therapeutics, AbbVie, Anthenex, OncoPep, Outcomes4Me, Kyowa Kirin Pharmaceuticals, Daiichi-Sankyo, Gilead and Samsung Bioepsis Inc. AW receives institutional research funding from Genentech/Roche.

The remaining authors declare that the research was conducted in the absence of any commercial or financial relationships that could be construed as a potential conflict of interest.

## References

[1] Hargadon KM Johnson CE Williams CJ. Immune Checkpoint Blockade Therapy for Cancer: An Overview of FDA-approved Immune Checkpoint Inhibitors. Int Immunopharmacol (2018) 62:29–39. 10.1016/j.intimp.2018.06.001 29990692

[2] Havel JJ Chowell D Chan TA. The Evolving Landscape of Biomarkers for Checkpoint Inhibitor Immunotherapy. Nat Rev Cancer (2019) 19(3):133–50. 10.1038/s41568-019-0116-x PMC6705396 30755690

[3] Chan TA Yarchoan M Jaffee E Swanton C Quezada SA Stenzinger A. Development of Tumor Mutation Burden as an Immunotherapy Biomarker: Utility for the Oncology Clinic. Ann Oncol (2019) 30(1):44–56. 10.1093/annonc/mdy495 30395155 PMC6336005

[4] Kandoth C McLellan MD Vandin F Ye K Niu B Lu C. Mutational Landscape and Significance Across 12 Major Cancer Types. Nature (2013) 502(7471):333–9. 10.1038/nature12634 PMC3927368 24132290

[5] Topalian SL Taube JM Anders RA Pardoll DM. Mechanism-Driven Biomarkers to Guide Immune Checkpoint Blockade in Cancer Therapy. Nat Rev Cancer (2016) 16(5):275–87. 10.1038/nrc.2016.36 PMC5381938 27079802

[6] Lawrence MS Stojanov P Polak P Kryukov GV Cibulskis K Sivachenko A. Mutational Heterogeneity in Cancer and the Search for New Cancer-Associated Genes. Nature (2013) 499(7457):214–8. 10.1038/nature12213 PMC3919509 23770567

[7] Ali HR Glont SE Blows FM Provenzano E Dawson SJ Liu B. Pd-L1 Protein Expression in Breast Cancer is Rare, Enriched in Basal-Like Tumours and Associated With Infiltrating Lymphocytes. Ann Oncol (2015) 26(7):1488–93. 10.1093/annonc/mdv192 25897014

[8] Wimberly H Brown JR Schalper K Haack H Silver MR Nixon C. Pd-L1 Expression Correlates With Tumor-Infiltrating Lymphocytes and Response to Neoadjuvant Chemotherapy in Breast Cancer. Cancer Immunol Res (2015) 3(4):326–32. 10.1158/2326-6066.CIR-14-0133 PMC4390454 25527356

[9] Thomas A Routh ED Pullikuth A Jin G Su J Chou JW. Tumor Mutational Burden is a Determinant of Immune-Mediated Survival in Breast Cancer. Oncoimmunology (2018) 7(10):e1490854. 10.1080/2162402X.2018.1490854 30386679 PMC6207420

[10] Denkert C von Minckwitz G Darb-Esfahani S Lederer B Heppner BI Weber KE. Tumour-Infiltrating Lymphocytes and Prognosis in Different Subtypes of Breast Cancer: A Pooled Analysis of 3771 Patients Treated With Neoadjuvant Therapy. Lancet Oncol (2018) 19(1):40–50. 10.1016/S1470-2045(17)30904-X 29233559

[11] Luen S Virassamy B Savas P Salgado R Loi S. The Genomic Landscape of Breast Cancer and its Interaction With Host Immunity. Breast (2016) 29:241–50. 10.1016/j.breast.2016.07.015 27481651

[12] Mittendorf EA Philips AV Meric-Bernstam F Qiao N Wu Y Harrington S. Pd-L1 Expression in Triple-Negative Breast Cancer. Cancer Immunol Res (2014) 2(4):361–70. 10.1158/2326-6066.CIR-13-0127 PMC4000553 24764583

[13] Nanda R Liu MC Yau C Shatsky R Pusztai L Wallace A. Effect of Pembrolizumab Plus Neoadjuvant Chemotherapy on Pathologic Complete Response in Women With Early-Stage Breast Cancer: An Analysis of the Ongoing Phase 2 Adaptively Randomized I-SPY2 Trial. JAMA Oncol (2020) 6(5):676–84. 10.1001/jamaoncol.2019.6650 PMC7058271 32053137

[14] Cortes J Cescon DW Rugo HS Nowecki Z Im SA Yusof MM. Pembrolizumab Plus Chemotherapy Versus Placebo Plus Chemotherapy for Previously Untreated Locally Recurrent Inoperable or Metastatic Triple-Negative Breast Cancer (KEYNOTE-355): A Randomised, Placebo-Controlled, Double-Blind, Phase 3 Clinical Trial. Lancet (2020) 396(10265):1817–28. 10.1016/S0140-6736(20)32531-9 33278935

[15] Schmid P Rugo HS Adams S Schneeweiss A Barrios CH Iwata H. Atezolizumab Plus Nab-Paclitaxel as First-Line Treatment for Unresectable, Locally Advanced or Metastatic Triple-Negative Breast Cancer (Impassion130): Updated Efficacy Results From a Randomised, Double-Blind, Placebo-Controlled, Phase 3 Trial. Lancet Oncol (2020) 21(1):44–59. 10.1016/S1470-2045(19)30689-8 31786121

[16] Schmid P Adams S Rugo HS Schneeweiss A Barrios CH Iwata H. Atezolizumab and Nab-Paclitaxel in Advanced Triple-Negative Breast Cancer. N Engl J Med (2018) 379(22):2108–21. 10.1056/NEJMoa1809615 30345906

[17] DeSantis CE Ma J Gaudet MM Newman LA Miller KD Goding Sauer A. Breast Cancer Statistics, 2019. CA: A Cancer J Clin (2019) 69(6):438–51. 10.3322/caac.21583 31577379

[18] Chung W Eum HH Lee HO Lee KM Lee HB Kim KT. Single-Cell RNA-seq Enables Comprehensive Tumour and Immune Cell Profiling in Primary Breast Cancer. Nat Commun (2017) 8:15081. 10.1038/ncomms15081 28474673 PMC5424158

[19] Loi S Sirtaine N Piette F Salgado R Viale G Van Eenoo F. Prognostic and Predictive Value of Tumor-Infiltrating Lymphocytes in a Phase III Randomized Adjuvant Breast Cancer Trial in Node-Positive Breast Cancer Comparing the Addition of Docetaxel to Doxorubicin With Doxorubicin-Based Chemotherapy: BIG 02-98. J Clin Oncol (2013) 31(7):860–7. 10.1200/JCO.2011.41.0902 23341518

[20] Thompson E Taube JM Elwood H Sharma R Meeker A Warzecha HN. The Immune Microenvironment of Breast Ductal Carcinoma in Situ. Mod Pathol (2016) 29(3):249–58. 10.1038/modpathol.2015.158 PMC5484584 26769139

[21] Schmid P Cortes J Pusztai L McArthur H Kummel S Bergh J. Pembrolizumab for Early Triple-Negative Breast Cancer. N Engl J Med (2020) 382(9):810–21. 10.1056/NEJMoa1910549 32101663

[22] Cortes J Cescon DW Rugo HS Nowecki Z Im S-A Yusof MM. Keynote-355: Randomized, Double-Blind, Phase III Study of Pembrolizumab + Chemotherapy Versus Placebo + Chemotherapy for Previously Untreated Locally Recurrent Inoperable or Metastatic Triple-Negative Breast Cancer. J Clin Oncol (2020) 38(15_suppl):1000–0. 10.1200/JCO.2020.38.15_suppl.1000 33278935

[23] Kurozumi S Inoue K Matsumoto H Fujii T Horiguchi J Oyama T. Clinicopathological Values of PD-L1 Expression in HER2-positive Breast Cancer. Sci Rep (2019) 9(1):16662. 10.1038/s41598-019-52944-6 31723167 PMC6853939

[24] Schalper KA Velcheti V Carvajal D Wimberly H Brown J Pusztai L. In Situ Tumor PD-L1 mRNA Expression is Associated With Increased TILs and Better Outcome in Breast Carcinomas. Clin Cancer Res (2014) 20(10):2773–82. 10.1158/1078-0432.CCR-13-2702 24647569

[25] Sobral-Leite M Van de Vijver K Michaut M van der Linden R Hooijer GKJ Horlings HM. Assessment of PD-L1 Expression Across Breast Cancer Molecular Subtypes, in Relation to Mutation Rate, BRCA1-like Status, Tumor-Infiltrating Immune Cells and Survival. Oncoimmunology (2018) 7(12):e1509820. 10.1080/2162402X.2018.1509820 30524905 PMC6279322

[26] Stanton SE Adams S Disis ML. Variation in the Incidence and Magnitude of Tumor-Infiltrating Lymphocytes in Breast Cancer Subtypes: A Systematic Review. JAMA Oncol (2016) 2(10):1354–60. 10.1001/jamaoncol.2016.1061 27355489

[27] Aaltomaa S Lipponen P Eskelinen M Kosma VM Marin S Alhava E. Lymphocyte Infiltrates as a Prognostic Variable in Female Breast Cancer. Eur J Cancer (1992) 28A(4-5):859–64. 10.1016/0959-8049(92)90134-n 1524909

[28] Savas P Salgado R Denkert C Sotiriou C Darcy PK Smyth MJ. Clinical Relevance of Host Immunity in Breast Cancer: From TILs to the Clinic. Nat Rev Clin Oncol (2016) 13(4):228–41. 10.1038/nrclinonc.2015.215 26667975

[29] Loi S Michiels S Salgado R Sirtaine N Jose V Fumagalli D. Tumor Infiltrating Lymphocytes are Prognostic in Triple Negative Breast Cancer and Predictive for Trastuzumab Benefit in Early Breast Cancer: Results From the FinHER Trial. Ann Oncol (2014) 25(8):1544–50. 10.1093/annonc/mdu112 24608200

[30] Dieci MV Mathieu MC Guarneri V Conte P Delaloge S Andre F. Prognostic and Predictive Value of Tumor-Infiltrating Lymphocytes in Two Phase III Randomized Adjuvant Breast Cancer Trials. Ann Oncol (2015) 26(8):1698–704. 10.1093/annonc/mdv239 PMC4511223 25995301

[31] Carbognin L Pilotto S Nortilli R Brunelli M Nottegar A Sperduti I. Predictive and Prognostic Role of Tumor-Infiltrating Lymphocytes for Early Breast Cancer According to Disease Subtypes: Sensitivity Analysis of Randomized Trials in Adjuvant and Neoadjuvant Setting. Oncologist (2016) 21(3):283–91. 10.1634/theoncologist.2015-0307 PMC4786352 26865589

[32] Krishnamurti U Wetherilt CS Yang J Peng L Li X. Tumor-Infiltrating Lymphocytes are Significantly Associated With Better Overall Survival and Disease-Free Survival in Triple-Negative But Not Estrogen Receptor-Positive Breast Cancers. Hum Pathol (2017) 64:7–12. 10.1016/j.humpath.2017.01.004 28153508

[33] Miyoshi Y Shien T Ogiya A Ishida N Yamazaki K Horli R. Associations in tumor infiltrating lymphocytes between clinicopathological factors and clinical outcomes in estrogen receptor-positive/human epidermal growth factor receptor type 2 negative breast cancer. Oncol Lett (2019) 17(2):2177–86. 10.3892/ol.2018.9853 PMC6341802 30675282

[34] Fujimoto Y Watanabe T Hida AI Higuchi T Miyagawa Y Ozawa H. Prognostic Significance of Tumor-Infiltrating Lymphocytes may Differ Depending on Ki67 Expression Levels in Estrogen Receptor-Positive/HER2-Negative Operated Breast Cancers. Breast Cancer (2019) 26(6):738–47. 10.1007/s12282-019-00977-0 31098866

[35] Ali HR Provenzano E Dawson SJ Blows FM Liu B Shah M. Association Between CD8+ T-Cell Infiltration and Breast Cancer Survival in 12,439 Patients. Ann Oncol (2014) 25(8):1536–43. 10.1093/annonc/mdu191 24915873

[36] Sobral-Leite M Salomon I Opdam M Kruger DT Beelen KJ van der Noort V. Cancer-Immune Interactions in ER-positive Breast Cancers: PI3K Pathway Alterations and Tumor-Infiltrating Lymphocytes. Breast Cancer Res (2019) 21(1):90. 10.1186/s13058-019-1176-2 31391067 PMC6686400

[37] Gu-Trantien C Loi S Garaud S Equeter C Libin M de Wind A. CD4(+) Follicular Helper T Cell Infiltration Predicts Breast Cancer Survival. J Clin Invest (2013) 123(7):2873–92. 10.1172/JCI67428 PMC3696556 23778140

[38] Liu S Foulkes WD Leung S Gao D Lau S Kos Z. Prognostic Significance of FOXP3+ Tumor-Infiltrating Lymphocytes in Breast Cancer Depends on Estrogen Receptor and Human Epidermal Growth Factor Receptor-2 Expression Status and Concurrent Cytotoxic T-cell Infiltration. Breast Cancer Res (2014) 16(5):432. 10.1186/s13058-014-0432-8 25193543 PMC4303113

[39] Koletsa T Kotoula V Koliou GA Manousou K Chrisafi S Zagouri F. Prognostic Impact of Stromal and Intratumoral CD3, CD8 and FOXP3 in Adjuvantly Treated Breast Cancer: do They Add Information Over Stromal Tumor-Infiltrating Lymphocyte Density? Cancer Immunol Immunother (2020) 69(8):1549–64. 10.1007/s00262-020-02557-0 PMC11027646 32303794

[40] Denkert C Loibl S Noske A Roller M Muller BM Komor M. Tumor-Associated Lymphocytes as an Independent Predictor of Response to Neoadjuvant Chemotherapy in Breast Cancer. J Clin Oncol (2010) 28(1):105–13. 10.1200/JCO.2009.23.7370 19917869

[41] Issa-Nummer Y Darb-Esfahani S Loibl S Kunz G Nekljudova V Schrader I. Prospective Validation of Immunological Infiltrate for Prediction of Response to Neoadjuvant Chemotherapy in HER2-negative Breast Cancer–a Substudy of the Neoadjuvant GeparQuinto Trial. PloS One (2013) 8(12):e79775. 10.1371/journal.pone.0079775 24312450 PMC3846472

[42] Skriver SK Jensen MB Knoop AS Ejlertsen B Laenkholm AV. Tumour-Infiltrating Lymphocytes and Response to Neoadjuvant Letrozole in Patients With Early Oestrogen Receptor-Positive Breast Cancer: Analysis From a Nationwide Phase II DBCG Trial. Breast Cancer Res (2020) 22(1):46. 10.1186/s13058-020-01285-8 32410705 PMC7222485

[43] Ono M Tsuda H Shimizu C Yamamoto S Shibata T Yamamoto H. Tumor-Infiltrating Lymphocytes are Correlated With Response to Neoadjuvant Chemotherapy in Triple-Negative Breast Cancer. Breast Cancer Res Treat (2012) 132(3):793–805. 10.1007/s10549-011-1554-7 21562709

[44] Hwang HW Jung H Hyeon J Park YH Ahn JS Im YH. A Nomogram to Predict Pathologic Complete Response (pCR) and the Value of Tumor-Infiltrating Lymphocytes (Tils) for Prediction of Response to Neoadjuvant Chemotherapy (NAC) in Breast Cancer Patients. Breast Cancer Res Treat (2019) 173(2):255–66. 10.1007/s10549-018-4981-x 30324273

[45] Russo L Maltese A Betancourt L Romero G Cialoni D De la Fuente L. Locally Advanced Breast Cancer: Tumor-infiltrating Lymphocytes as a Predictive Factor of Response to Neoadjuvant Chemotherapy. Eur J Surg Oncol (2019) 45(6):963–8. 10.1016/j.ejso.2019.01.222 30745134

[46] Ali HR Dariush A Thomas J Provenzano E Dunn J Hiller L. Lymphocyte Density Determined by Computational Pathology Validated as a Predictor of Response to Neoadjuvant Chemotherapy in Breast Cancer: Secondary Analysis of the ARTemis Trial. Ann Oncol (2017) 28(8):1832–5. 10.1093/annonc/mdx266 PMC5834010 28525534

[47] Seo AN Lee HJ Kim EJ Kim HJ Jang MH Lee HE. Tumour-Infiltrating CD8+ Lymphocytes as an Independent Predictive Factor for Pathological Complete Response to Primary Systemic Therapy in Breast Cancer. Br J Cancer (2013) 109(10):2705–13. 10.1038/bjc.2013.634 PMC3833219 24129232

[48] Brown JR Wimberly H Lannin DR Nixon C Rimm DL Bossuyt V. Multiplexed Quantitative Analysis of CD3, CD8, and CD20 Predicts Response to Neoadjuvant Chemotherapy in Breast Cancer. Clin Cancer Res (2014) 20(23):5995–6005. 10.1158/1078-0432.CCR-14-1622 25255793 PMC4252785

[49] Watanabe T Hida AI Inoue N Imamura M Fujimoto Y Akazawa K. Abundant Tumor Infiltrating Lymphocytes After Primary Systemic Chemotherapy Predicts Poor Prognosis in Estrogen Receptor-Positive/HER2-Negative Breast Cancers. Breast Cancer Res Treat (2018) 168(1):135–45. 10.1007/s10549-017-4575-z 29168063

[50] Pelekanou V Carvajal-Hausdorf DE Altan M Wasserman B Carvajal-Hausdorf C Wimberly H. Effect of Neoadjuvant Chemotherapy on Tumor-Infiltrating Lymphocytes and PD-L1 Expression in Breast Cancer and its Clinical Significance. Breast Cancer Res (2017) 19(1):91. 10.1186/s13058-017-0884-8 28784153 PMC5547502

[51] Hamy AS Bonsang-Kitzis H De Croze D Laas E Darrigues L Topciu L. Interaction Between Molecular Subtypes and Stromal Immune Infiltration Before and After Treatment in Breast Cancer Patients Treated With Neoadjuvant Chemotherapy. Clin Cancer Res (2019) 25(22):6731–41. 10.1158/1078-0432.CCR-18-3017 31515462

[52] Ladoire S Mignot G Dabakuyo S Arnould L Apetoh L Rebe C. In Situ Immune Response After Neoadjuvant Chemotherapy for Breast Cancer Predicts Survival. J Pathol (2011) 224(3):389–400. 10.1002/path.2866 21437909

[53] Asano Y Kashiwagi S Goto W Takada K Takahashi K Hatano T. Prediction of Survival After Neoadjuvant Chemotherapy for Breast Cancer by Evaluation of Tumor-Infiltrating Lymphocytes and Residual Cancer Burden. BMC Cancer (2017) 17(1):888. 10.1186/s12885-017-3927-8 29282021 PMC5745786

[54] Salgado R Denkert C Demaria S Sirtaine N Klauschen F Pruneri G. The Evaluation of Tumor-Infiltrating Lymphocytes (Tils) in Breast Cancer: Recommendations by an International Tils Working Group 2014. Ann Oncol (2015) 26(2):259–71. 10.1093/annonc/mdu450 PMC6267863 25214542

[55] Denkert C Wienert S Poterie A Loibl S Budczies J Badve S. Standardized Evaluation of Tumor-Infiltrating Lymphocytes in Breast Cancer: Results of the Ring Studies of the International Immuno-Oncology Biomarker Working Group. Mod Pathol (2016) 29(10):1155–64. 10.1038/modpathol.2016.109 27363491

[56] Cortazar P Zhang L Untch M Mehta K Costantino JP Wolmark N. Pathological Complete Response and Long-Term Clinical Benefit in Breast Cancer: The CTNeoBC Pooled Analysis. Lancet (2014) 384(9938):164–72. 10.1016/S0140-6736(13)62422-8 24529560

[57] Symmans WF Wei C Gould R Yu X Zhang Y Liu M. Long-Term Prognostic Risk After Neoadjuvant Chemotherapy Associated With Residual Cancer Burden and Breast Cancer Subtype. J Clin Oncol (2017) 35(10):1049–60. 10.1200/JCO.2015.63.1010 PMC5455352 28135148

[58] von Minckwitz G Raab G Caputo A Schutte M Hilfrich J Blohmer JU. Doxorubicin With Cyclophosphamide Followed by Docetaxel Every 21 Days Compared With Doxorubicin and Docetaxel Every 14 Days as Preoperative Treatment in Operable Breast Cancer: The GEPARDUO Study of the German Breast Group. J Clin Oncol (2005) 23(12):2676–85. 10.1200/JCO.2005.05.078 15837982

[59] von Minckwitz G Kummel S Vogel P Hanusch C Eidtmann H Hilfrich J. Neoadjuvant Vinorelbine-Capecitabine Versus Docetaxel-Doxorubicin-Cyclophosphamide in Early Nonresponsive Breast Cancer: Phase III Randomized GeparTrio Trial. J Natl Cancer Inst (2008) 100(8):542–51. 10.1093/jnci/djn085 18398097

[60] Waks AG Stover DG Guerriero JL Dillon D Barry WT Gjini E. The Immune Microenvironment in Hormone Receptor-Positive Breast Cancer Before and After Preoperative Chemotherapy. Clin Cancer Res (2019) 25(15):4644–55. 10.1158/1078-0432.CCR-19-0173 PMC6677598 31061067

[61] Zhang L Wang XI Zhang S. Tumor-Infiltrating Lymphocyte Volume is a Better Predictor of Neoadjuvant Therapy Response and Overall Survival in Triple-Negative Invasive Breast Cancer. Hum Pathol (2018) 80:47–54. 10.1016/j.humpath.2018.05.024 29883779

[62] Ruffell B Au A Rugo HS Esserman LJ Hwang ES Coussens LM. Leukocyte Composition of Human Breast Cancer. Proc Natl Acad Sci USA (2012) 109(8):2796–801. 10.1073/pnas.1104303108 PMC3287000 21825174

[63] Dieci MV Radosevic-Robin N Fineberg S van den Eynden G Ternes N Penault-Llorca F. Update on Tumor-Infiltrating Lymphocytes (Tils) in Breast Cancer, Including Recommendations to Assess TILs in Residual Disease After Neoadjuvant Therapy and in Carcinoma in Situ: A Report of the International Immuno-Oncology Biomarker Working Group on Breast Cancer. Semin Cancer Biol (2018) 52(Pt 2):16–25. 10.1016/j.semcancer.2017.10.003 29024776

[64] Dieci MV Criscitiello C Goubar A Viale G Conte P Guarneri V. Prognostic Value of Tumor-Infiltrating Lymphocytes on Residual Disease After Primary Chemotherapy for Triple-Negative Breast Cancer: A Retrospective Multicenter Study. Ann Oncol (2014) 25(3):611–8. 10.1093/annonc/mdt556 PMC3933248 24401929

[65] Dieci MV Frassoldati A Generali D Bisagni G Piacentini F Cavanna L. Tumor-Infiltrating Lymphocytes and Molecular Response After Neoadjuvant Therapy for HR+/HER2- Breast Cancer: Results From Two Prospective Trials. Breast Cancer Res Treat (2017) 163(2):295–302. 10.1007/s10549-017-4191-y 28289852

[66] Kotoula V Chatzopoulos K Lakis S Alexopoulou Z Timotheadou E Zagouri F. Tumors With High-Density Tumor Infiltrating Lymphocytes Constitute a Favorable Entity in Breast Cancer: A Pooled Analysis of Four Prospective Adjuvant Trials. Oncotarget (2016) 7(4):5074–87. 10.18632/oncotarget.6231 PMC4826267 26506242

[67] Gruosso T Gigoux M Manem VSK Bertos N Zuo D Perlitch I. Spatially Distinct Tumor Immune Microenvironments Stratify Triple-Negative Breast Cancers. J Clin Invest (2019) 129(4):1785–800. 10.1172/JCI96313 PMC6436884 30753167

[68] Bareche Y Buisseret L Gruosso T Girard E Venet D Dupont F. Unraveling Triple-Negative Breast Cancer Tumor Microenvironment Heterogeneity: Towards an Optimized Treatment Approach. JNCI: J Natl Cancer Institute (2019) 112(7):708–19. 10.1093/jnci/djz208 PMC7357326 31665482

[69] Li X Gruosso T Zuo D Omeroglu A Meterissian S Guiot M-C. Infiltration of CD8+ T Cells Into Tumor Cell Clusters in Triple-Negative Breast Cancer. Proc Natl Acad Sci (2019) 116(9):3678–87. 10.1073/pnas.1817652116 PMC6397588 30733298

[70] Sautes-Fridman C Lawand M Giraldo NA Kaplon H Germain C Fridman WH. Tertiary Lymphoid Structures in Cancers: Prognostic Value, Regulation, and Manipulation for Therapeutic Intervention. Front Immunol (2016) 7:407. 10.3389/fimmu.2016.00407 27752258 PMC5046074

[71] Colbeck EJ Ager A Gallimore A Jones GW. Tertiary Lymphoid Structures in Cancer: Drivers of Antitumor Immunity, Immunosuppression, or Bystander Sentinels in Disease? Front Immunol (2017) 8:1830. 10.3389/fimmu.2017.01830 29312327 PMC5742143

[72] Dieu-Nosjean MC Goc J Giraldo NA Sautès-Fridman C Fridman WH. Tertiary Lymphoid Structures in Cancer and Beyond. Trends Immunol (2014) 35(11):571–80. 10.1016/j.it.2014.09.006 25443495

[73] Cabrita R Lauss M Sanna A Donia M Skaarup Larsen M Mitra S. Tertiary Lymphoid Structures Improve Immunotherapy and Survival in Melanoma. Nature (2020) 577(7791):561–5. 10.1038/s41586-019-1914-8 31942071

[74] Lee HJ Park IA Song IH Shin SJ Kim JY Yu JH. Tertiary Lymphoid Structures: Prognostic Significance and Relationship With Tumour-Infiltrating Lymphocytes in Triple-Negative Breast Cancer. J Clin Pathol (2016) 69(5):422–30. 10.1136/jclinpath-2015-203089 26475777

[75] Lee M Heo SH Song IH Rajayi H Park HS Park IA. Presence of Tertiary Lymphoid Structures Determines the Level of Tumor-Infiltrating Lymphocytes in Primary Breast Cancer and Metastasis. Mod Pathol (2019) 32(1):70–80. 10.1038/s41379-018-0113-8 30154578

[76] Liu X Tsang JYS Hlaing T Hu J Ni YB Chan SK. Distinct Tertiary Lymphoid Structure Associations and Their Prognostic Relevance in HER2 Positive and Negative Breast Cancers. Oncologist (2017) 22(11):1316–24. 10.1634/theoncologist.2017-0029 PMC5679825 28701569

[77] Petitprez F de Reynies A Keung EZ Chen TW Sun CM Calderaro J. B Cells are Associated With Survival and Immunotherapy Response in Sarcoma. Nature (2020) 577(7791):556–60. 10.1038/s41586-019-1906-8 31942077

[78] Song IH Heo SH Bang WS Park HS Park IA Kim YA. Predictive Value of Tertiary Lymphoid Structures Assessed by High Endothelial Venule Counts in the Neoadjuvant Setting of Triple-Negative Breast Cancer. Cancer Res Treat (2017) 49(2):399–407. 10.4143/crt.2016.215 27488875 PMC5398384

[79] Martinet L Garrido I Filleron T Le Guellec S Bellard E Fournie JJ. Human Solid Tumors Contain High Endothelial Venules: Association With T- and B-lymphocyte Infiltration and Favorable Prognosis in Breast Cancer. Cancer Res (2011) 71(17):5678–87. 10.1158/0008-5472.CAN-11-0431 21846823

[80] Sofopoulos M Fortis SP Vaxevanis CK Sotiriadou NN Arnogiannaki N Ardavanis A. The Prognostic Significance of Peritumoral Tertiary Lymphoid Structures in Breast Cancer. Cancer Immunol Immunother (2019) 68(11):1733–45. 10.1007/s00262-019-02407-8 PMC11028375 31598757

[81] Buisseret L Desmedt C Garaud S Fornili M Wang X Van den Eyden G. Reliability of Tumor-Infiltrating Lymphocyte and Tertiary Lymphoid Structure Assessment in Human Breast Cancer. Mod Pathol (2017) 30(9):1204–12. 10.1038/modpathol.2017.43 28621322

[82] Helmink BA Reddy SM Gao J Zhang S Basar R Thakur R. B Cells and Tertiary Lymphoid Structures Promote Immunotherapy Response. Nature (2020) 577(7791):549–55. 10.1038/s41586-019-1922-8 PMC8762581 31942075

[83] Leone P Shin EC Perosa F Vacca A Dammacco F Racanelli V. MHC Class I Antigen Processing and Presenting Machinery: Organization, Function, and Defects in Tumor Cells. J Natl Cancer Inst (2013) 105(16):1172–87. 10.1093/jnci/djt184 23852952

[84] Kaneko K Ishigami S Kijima Y Funasako Y Hirata M Okumura H. Clinical Implication of HLA Class I Expression in Breast Cancer. BMC Cancer (2011) 11:454. 10.1186/1471-2407-11-454 22014037 PMC3214195

[85] Kikuchi E Yamazaki K Torigoe T Cho Y Miyamoto M Oizumi S. HLA Class I Antigen Expression is Associated With a Favorable Prognosis in Early Stage non-Small Cell Lung Cancer. Cancer Sci (2007) 98(9):1424–30. 10.1111/j.1349-7006.2007.00558.x PMC11159758 17645781

[86] Menon AG Morreau H Tollenaar RA Alphenaar E Van Puijenbroek M Putter H. Down-Regulation of HLA-A Expression Correlates With a Better Prognosis in Colorectal Cancer Patients. Lab Invest (2002) 82(12):1725–33. 10.1097/01.lab.0000043124.75633.ed 12480922

[87] Kitamura H Honma I Torigoe T Asanuma H Sato N Tsukamoto T. Down-Regulation of HLA Class I Antigen is an Independent Prognostic Factor for Clear Cell Renal Cell Carcinoma. J Urol (2007) 177(4):1269–72; discussion 1272. 10.1016/j.juro.2006.11.082 17382705

[88] Rusakiewicz S Semeraro M Sarabi M Desbois M Locher C Mendez R. Immune Infiltrates are Prognostic Factors in Localized Gastrointestinal Stromal Tumors. Cancer Res (2013) 73(12):3499–510. 10.1158/0008-5472.CAN-13-0371 23592754

[89] Tsukahara T Kawaguchi S Torigoe T Asanuma H Nakazawa E Shimozawa K. Prognostic Significance of HLA Class I Expression in Osteosarcoma Defined by Anti-Pan HLA Class I Monoclonal Antibody, EMR8-5. Cancer Sci (2006) 97(12):1374–80. 10.1111/j.1349-7006.2006.00317.x PMC11158095 16995877

[90] Garrido F Ruiz-Cabello F Cabrera T Perez-Villar JJ Lopez-Botet M Duggan-Keen M. Implications for Immunosurveillance of Altered HLA Class I Phenotypes in Human Tumours. Immunol Today (1997) 18(2):89–95. 10.1016/s0167-5699(96)10075-x 9057360

[91] Torigoe T Asanuma H Nakazawa E Tamura Y Hirohashi Y Yamamoto E. Establishment of a Monoclonal Anti-Pan HLA Class I Antibody Suitable for Immunostaining of Formalin-Fixed Tissue: Unusually High Frequency of Down-Regulation in Breast Cancer Tissues. Pathol Int (2012) 62(5):303–8. 10.1111/j.1440-1827.2012.02789.x 22524657

[92] Garrido MA Rodriguez T Zinchenko S Maleno I Ruiz-Cabello F Concha A. HLA Class I Alterations in Breast Carcinoma are Associated With a High Frequency of the Loss of Heterozygosity At Chromosomes 6 and 15. Immunogenetics (2018) 70(10):647–59. 10.1007/s00251-018-1074-2 30145665

[93] Sinn BV Weber KE Schmitt WD Fasching PA Symmans WF Blohmer JU. Human Leucocyte Antigen Class I in Hormone Receptor-Positive, HER2-negative Breast Cancer: Association With Response and Survival After Neoadjuvant Chemotherapy. Breast Cancer Res (2019) 21(1):142. 10.1186/s13058-019-1231-z 31829264 PMC6907189

[94] Lee HJ Song IH Park IA Heo SH Kim YA Ahn JH. Differential Expression of Major Histocompatibility Complex Class I in Subtypes of Breast Cancer is Associated With Estrogen Receptor and Interferon Signaling. Oncotarget (2016) 7(21):30119–32. 10.18632/oncotarget.8798 PMC5058668 27121061

[95] Kaklamanis L Leek R Koukourakis M Gatter KC Harris AL. Loss of Transporter in Antigen Processing 1 Transport Protein and Major Histocompatibility Complex Class I Molecules in Metastatic Versus Primary Breast Cancer. Cancer Res (1995) 55(22):5191–4. 7585572

[96] Liu Y Komohara Y Domenick N Ohno M Ikeura M Hamilton RL. Expression of Antigen Processing and Presenting Molecules in Brain Metastasis of Breast Cancer. Cancer Immunol Immunother (2012) 61(6):789–801. 10.1007/s00262-011-1137-9 22065046 PMC3365630

[97] Park HS Cho U Im SY Yoo CY Jung JH Suh YJ. Loss of Human Leukocyte Antigen Class I Expression Is Associated With Poor Prognosis in Patients With Advanced Breast Cancer. J Pathol Transl Med (2019) 53(2):75–85. 10.4132/jptm.2018.10.11 30424591 PMC6435992

[98] Madjd Z Spendlove I Pinder SE Ellis IO Durrant LG. Total Loss of MHC Class I is an Independent Indicator of Good Prognosis in Breast Cancer. Int J Cancer (2005) 117(2):248–55. 10.1002/ijc.21163 15900607

[99] Garrido F Cabrera T Aptsiauri N. “Hard” and “Soft” Lesions Underlying the HLA Class I Alterations in Cancer Cells: Implications for Immunotherapy. Int J Cancer (2010) 127(2):249–56. 10.1002/ijc.25270 20178101

[100] Shimasaki N Jain A Campana D. NK Cells for Cancer Immunotherapy. Nat Rev Drug Discovery (2020) 19(3):200–18. 10.1038/s41573-019-0052-1 31907401

[101] Miller JS Lanier LL. Natural Killer Cells in Cancer Immunotherapy. Annu Rev Cancer Biol (2019) 3(1):77–103. 10.1146/annurev-cancerbio-030518-055653

[102] Solinas C Carbognin L De Silva P Criscitiello C Lambertini M. Tumor-Infiltrating Lymphocytes in Breast Cancer According to Tumor Subtype: Current State of the Art. Breast (2017) 35:142–50. 10.1016/j.breast.2017.07.005 28735162

[103] Jiang J Pan W Xu Y Ni C Xue D Chen Z. Tumour-Infiltrating Immune Cell-Based Subtyping and Signature Gene Analysis in Breast Cancer Based on Gene Expression Profiles. J Cancer (2020) 11(6):1568–83. 10.7150/jca.37637 PMC6995381 32047563

[104] Liu Z Li M Jiang Z Wang X. A Comprehensive Immunologic Portrait of Triple-Negative Breast Cancer. Transl Oncol (2018) 11(2):311–29. 10.1016/j.tranon.2018.01.011 PMC5884188 29413765

[105] O’Meara T Marczyk M Qing T Yaghoobi V Blenman K Cole K. Immunological Differences Between Immune-Rich Estrogen Receptor-Positive and Immune-Rich Triple-Negative Breast Cancers. JCO Precis Oncol (2020) 4:767–79. 10.1200/po.19.00350 PMC7446500 32923897

[106] Frazao A Messaoudene M Nunez N Dulphy N Roussin F Sedlik C. CD16(+)NKG2A(High) Natural Killer Cells Infiltrate Breast Cancer-Draining Lymph Nodes. Cancer Immunol Res (2019) 7(2):208–18. 10.1158/2326-6066.Cir-18-0085 30514793

[107] Cooley S Burns LJ Repka T Miller JS. Natural Killer Cell Cytotoxicity of Breast Cancer Targets is Enhanced by Two Distinct Mechanisms of Antibody-Dependent Cellular Cytotoxicity Against LFA-3 and HER2/Neu. Exp Hematol (1999) 27(10):1533–41. 10.1016/s0301-472x(99)00089-2 10517495

[108] Kajitani K Tanaka Y Arihiro K Kataoka T Ohdan H. Mechanistic Analysis of the Antitumor Efficacy of Human Natural Killer Cells Against Breast Cancer Cells. Breast Cancer Res Treat (2012) 134(1):139–55. 10.1007/s10549-011-1944-x 22261932

[109] Schönfeld K Sahm C Zhang C Naundorf S Brendel C Odendahl M. Selective Inhibition of Tumor Growth by Clonal NK Cells Expressing an ErbB2/HER2-specific Chimeric Antigen Receptor. Mol Ther (2015) 23(2):330–8. 10.1038/mt.2014.219 PMC4445620 25373520

[110] Liu H Yang B Sun T Lin L Hu Y Deng M. Specific Growth Inhibition of ErbB2−expressing Human Breast Cancer Cells by Genetically Modified NK−92 Cells. Oncol Rep (2015) 33(1):95–102. 10.3892/or.2014.3548 25333815

[111] Hu Z. Tissue Factor as a New Target for CAR-NK Cell Immunotherapy of Triple-Negative Breast Cancer. Sci Rep (2020) 10(1):2815. 10.1038/s41598-020-59736-3 32071339 PMC7028910

[112] Sahm C Schönfeld K Wels WS. Expression of IL-15 in NK Cells Results in Rapid Enrichment and Selective Cytotoxicity of Gene-Modified Effectors That Carry a Tumor-Specific Antigen Receptor. Cancer Immunol Immunother (2012) 61(9):1451–61. 10.1007/s00262-012-1212-x PMC11029748 22310931

[113] Chen X Han J Chu J Zhang L Zhang J Chen C. A Combinational Therapy of EGFR-CAR NK Cells and Oncolytic Herpes Simplex Virus 1 for Breast Cancer Brain Metastases. Oncotarget (2016) 7(19):27764–77. 10.18632/oncotarget.8526 PMC5053686 27050072

[114] Osta WA Chen Y Mikhitarian K Mitas M Salem M Hannun YA. EpCAM is Overexpressed in Breast Cancer and is a Potential Target for Breast Cancer Gene Therapy. Cancer Res (2004) 64(16):5818–24. 10.1158/0008-5472.Can-04-0754 15313925

[115] Muntasell A Cabo M Servitja S Tusquets I Martínez-García M Rovira A. Interplay Between Natural Killer Cells and Anti-HER2 Antibodies: Perspectives for Breast Cancer Immunotherapy. Front Immunol (2017) 8:1544. 10.3389/fimmu.2017.01544 29181007 PMC5694168

[116] Binnewies M Roberts EW Kersten K Chan V Fearon DF Merad M. Understanding the Tumor Immune Microenvironment (TIME) for Effective Therapy. Nat Med (2018) 24(5):541–50. 10.1038/s41591-018-0014-x PMC5998822 29686425

[117] Ali HR Chlon L Pharoah PD Markowetz F Caldas C. Patterns of Immune Infiltration in Breast Cancer and Their Clinical Implications: A Gene-Expression-Based Retrospective Study. PloS Med (2016) 13(12):e1002194. 10.1371/journal.pmed.1002194 27959923 PMC5154505

[118] Bense RD Sotiriou C Piccart-Gebhart MJ Haanen J van Vugt M de Vries EGE. Relevance of Tumor-Infiltrating Immune Cell Composition and Functionality for Disease Outcome in Breast Cancer. J Natl Cancer Inst (2017) 109(1):djw192. 10.1093/jnci/djw192 PMC6284248 27737921

[119] Condeelis J Pollard JW. Macrophages: Obligate Partners for Tumor Cell Migration, Invasion, and Metastasis. Cell (2006) 124(2):263–6. 10.1016/j.cell.2006.01.007 16439202

[120] Lan C Huang X Lin S Huang H Cai Q Wan T. Expression of M2-polarized Macrophages is Associated With Poor Prognosis for Advanced Epithelial Ovarian Cancer. Technol Cancer Res Treat (2013) 12(3):259–67. 10.7785/tcrt.2012.500312 23289476

[121] Medrek C Ponten F Jirstrom K Leandersson K. The Presence of Tumor Associated Macrophages in Tumor Stroma as a Prognostic Marker for Breast Cancer Patients. BMC Cancer (2012) 12:306. 10.1186/1471-2407-12-306 22824040 PMC3414782

[122] Qian BZ Pollard JW. Macrophage Diversity Enhances Tumor Progression and Metastasis. Cell (2010) 141(1):39–51. 10.1016/j.cell.2010.03.014 20371344 PMC4994190

[123] Solinas G Germano G Mantovani A Allavena P. Tumor-Associated Macrophages (TAM) as Major Players of the Cancer-Related Inflammation. J Leukoc Biol (2009) 86(5):1065–73. 10.1189/jlb.0609385 19741157

[124] Tymoszuk P Charoentong P Hackl H Spilka R Muller-Holzner E Trajanoski Z. High STAT1 mRNA Levels But Not its Tyrosine Phosphorylation are Associated With Macrophage Infiltration and Bad Prognosis in Breast Cancer. BMC Cancer (2014) 14:257. 10.1186/1471-2407-14-257 24725474 PMC4021106

[125] Guerriero JL. Macrophages: The Road Less Traveled, Changing Anticancer Therapy. Trends Mol Med (2018) 24(5):472–89. 10.1016/j.molmed.2018.03.006 PMC5927840 29655673

[126] Castellaro AM Rodriguez-Baili MC Di Tada CE Gil GA. Tumor-Associated Macrophages Induce Endocrine Therapy Resistance in ER+ Breast Cancer Cells. Cancers (Basel) (2019) 11(2):189. 10.3390/cancers11020189 PMC6406935 30736340

[127] Mehta AK Kadel S Townsend MG Oliwa M Guerriero JL. Macrophage Biology and Mechanisms of Immune Suppression in Breast Cancer. Front Immunol (2021) 10.3389/fimmu.2021.643771 PMC8102870 33968034

[128] Wesolowski R Sharma N Reebel L Rodal MB Peck A West BL. Phase Ib Study of the Combination of Pexidartinib (PLX3397), a CSF-1R Inhibitor, and Paclitaxel in Patients With Advanced Solid Tumors. Ther Adv Med Oncol (2019) 11:1758835919854238. 10.1177/1758835919854238 31258629 PMC6589951

[129] Allison JP McIntyre BW Bloch D. Tumor-Specific Antigen of Murine T-lymphoma Defined With Monoclonal Antibody. J Immunol (1982) 129(5):2293–300. 6181166

[130] Krummel MF Allison JP. CD28 and CTLA-4 Have Opposing Effects on the Response of T Cells to Stimulation. J Exp Med (1995) 182(2):459–65. 10.1084/jem.182.2.459 PMC2192127 7543139

[131] Leach DR Krummel MF Allison JP. Enhancement of Antitumor Immunity by CTLA-4 Blockade. Science (1996) 271(5256):1734–6. 10.1126/science.271.5256.1734 8596936

[132] Nishimura H Nose M Hiai H Minato N Honjo T. Development of Lupus-Like Autoimmune Diseases by Disruption of the PD-1 Gene Encoding an ITIM Motif-Carrying Immunoreceptor. Immunity (1999) 11(2):141–51. 10.1016/s1074-7613(00)80089-8 10485649

[133] Butte MJ Keir ME Phamduy TB Sharpe AH Freeman GJ. Programmed Death-1 Ligand 1 Interacts Specifically With the B7-1 Costimulatory Molecule to Inhibit T Cell Responses. Immunity (2007) 27(1):111–22. 10.1016/j.immuni.2007.05.016 PMC2707944 17629517

[134] Gatalica Z Snyder C Maney T Ghazalpour A Holterman DA Xiao N. Programmed Cell Death 1 (PD-1) and its Ligand (PD-L1) in Common Cancers and Their Correlation With Molecular Cancer Type. Cancer Epidemiol Biomarkers Prev (2014) 23(12):2965–70. 10.1158/1055-9965.EPI-14-0654 25392179

[135] Sharma P Hu-Lieskovan S Wargo JA Ribas A. Primary, Adaptive, and Acquired Resistance to Cancer Immunotherapy. Cell (2017) 168(4):707–23. 10.1016/j.cell.2017.01.017 PMC5391692 28187290

[136] Sabatier R Finetti P Mamessier E Adelaide J Chaffanet M Ali HR. Prognostic and Predictive Value of PDL1 Expression in Breast Cancer. Oncotarget (2015) 6(7):5449–64. 10.18632/oncotarget.3216 PMC4467160 25669979

[137] Beckers RK Selinger CI Vilain R Madore J Wilmott JS Harvey K. Programmed Death Ligand 1 Expression in Triple-Negative Breast Cancer is Associated With Tumour-Infiltrating Lymphocytes and Improved Outcome. Histopathology (2016) 69(1):25–34. 10.1111/his.12904 26588661

[138] Mardones M Grosser D Levin M Daoud Y Palucka K O’Shaughnessy J. Abstract P2-04-20: Pd-L1 Expression in Triple Negative Breast Cancer (TNBC) is Associated With Improved Outcomes. Cancer Res (2017) 77(4 Supplement):P2–04-20-P2-04-20. 10.1158/1538-7445.Sabcs16-p2-04-20

[139] Zerdes I Sifakis EG Matikas A Chretien S Tobin NP Hartman J. Programmed Death-Ligand 1 Gene Expression is a Prognostic Marker in Early Breast Cancer and Provides Additional Prognostic Value to 21-Gene and 70-Gene Signatures in Estrogen Receptor-Positive Disease. Mol Oncol (2020) 14(5):951–63. 10.1002/1878-0261.12654 PMC7191187 32115850

[140] Rugo HS Delord JP Im SA Ott PA Piha-Paul SA Bedard PL. Safety and Antitumor Activity of Pembrolizumab in Patients With Estrogen Receptor-Positive/Human Epidermal Growth Factor Receptor 2-Negative Advanced Breast Cancer. Clin Cancer Res (2018) 24(12):2804–11. 10.1158/1078-0432.CCR-17-3452 29559561

[141] Nanda R Chow LQ Dees EC Berger R Gupta S Geva R. Pembrolizumab in Patients With Advanced Triple-Negative Breast Cancer: Phase Ib KEYNOTE-012 Study. J Clin Oncol (2016) 34(21):2460–7. 10.1200/JCO.2015.64.8931 PMC6816000 27138582

[142] Marra A Viale G Curigliano G. Recent Advances in Triple Negative Breast Cancer: The Immunotherapy Era. BMC Med (2019) 17(1):90. 10.1186/s12916-019-1326-5 31068190 PMC6507064

[143] Dirix LY Takacs I Jerusalem G Nikolinakos P Arkenau HT Forero-Torres A. Avelumab, an anti-PD-L1 Antibody, in Patients With Locally Advanced or Metastatic Breast Cancer: A Phase 1b JAVELIN Solid Tumor Study. Breast Cancer Res Treat (2018) 167(3):671–86. 10.1007/s10549-017-4537-5 PMC5807460 29063313

[144] Rasmussen L Arvin A. Chemotherapy-Induced Immunosuppression. Environ Health Perspect (1982) 43:21–5. 10.1289/ehp.824321 PMC1568884 7037385

[145] Ramakrishnan R Assudani D Nagaraj S Hunter T Cho HI Antonia S. Chemotherapy Enhances Tumor Cell Susceptibility to CTL-mediated Killing During Cancer Immunotherapy in Mice. J Clin Invest (2010) 120(4):1111–24. 10.1172/JCI40269 PMC2846048 20234093

[146] Kaneno R Shurin GV Kaneno FM Naiditch H Luo J Shurin MR. Chemotherapeutic Agents in Low Noncytotoxic Concentrations Increase Immunogenicity of Human Colon Cancer Cells. Cell Oncol (Dordr) (2011) 34(2):97–106. 10.1007/s13402-010-0005-5 21290210 PMC13014597

[147] Emens LA Braiteh FS Cassier F Delord J-P Eder JP Fasso M. Inhibition of PD-L1 by MPDL3280A Leads to Clinical Activity in Patients With Metastatic Triple-Negative Breast Cancer (TNBC). Cancer Res (2015) 75:2859. 10.1158/1538-7445.AM2015-2859

[148] Nanda R Chow LQ Dees EC Berger R Gupta S Geva R. A Phase Ib Study of Pembrolizumab (MK-3475) in Patients With Advanced Triple-Negative Breast Cancer. Cancer Res (2015) 75. 10.1158/1538-7445.SABCS14-S1-09

[149] Cortes J Schoffski P Littlefield BA. Multiple Modes of Action of Eribulin Mesylate: Emerging Data and Clinical Implications. Cancer Treat Rev (2018) 70:190–8. 10.1016/j.ctrv.2018.08.008 30243063

[150] Goto W Kashiwagi S Asano Y Takada K Morisaki T Fujita H. Eribulin Promotes Antitumor Immune Responses in Patients With Locally Advanced or Metastatic Breast Cancer. Anticancer Res (2018) 38(5):2929–38. 10.21873/anticanres.12541 29715119

[151] Tolaney SM Barroso-Sousa R Keenan T Trippa L Hu J Luis IMVD. Randomized Phase II Study of Eribulin Mesylate (E) With or Without Pembrolizumab (P) for Hormone Receptor-Positive (HR+) Metastatic Breast Cancer (MBC). J Clin Oncol (2019) 37(15_suppl):1004–4. 10.1200/JCO.2019.37.15_suppl.1004

[152] Shah AN Flaum L Helenowski I Santa-Maria CA Jain S Rademaker A. Phase II Study of Pembrolizumab and Capecitabine for Triple Negative and Hormone Receptor-Positive, HER2-negative Endocrine-Refractory Metastatic Breast Cancer. J Immunother Cancer (2020) 8(1):e000173. 10.1136/jitc-2019-000173 32060053 PMC7057426

[153] Correale P Aquino A Giuliani A Pellegrini M Micheli L Cusi MG. Treatment of Colon and Breast Carcinoma Cells With 5-Fluorouracil Enhances Expression of Carcinoembryonic Antigen and Susceptibility to HLA-A(*)02.01 Restricted, CEA-peptide-specific Cytotoxic T Cells In Vitro. Int J Cancer (2003) 104(4):437–45. 10.1002/ijc.10969 12584740

[154] Vincent J Mignot G Chalmin F Ladoire S Bruchard M Chevriaux A. 5-Fluorouracil Selectively Kills Tumor-Associated Myeloid-Derived Suppressor Cells Resulting in Enhanced T Cell-Dependent Antitumor Immunity. Cancer Res (2010) 70(8):3052–61. 10.1158/0008-5472.CAN-09-3690 20388795

[155] Annels NE Shaw VE Gabitass RF Billingham L Corrie P Eatock M. The Effects of Gemcitabine and Capecitabine Combination Chemotherapy and of Low-Dose Adjuvant GM-CSF on the Levels of Myeloid-Derived Suppressor Cells in Patients With Advanced Pancreatic Cancer. Cancer Immunol Immunother (2014) 63(2):175–83. 10.1007/s00262-013-1502-y PMC11028876 24292263

[156] Middleton G Greenhalf W Costello E Shaw V Cox T Ghaneh P. Immunobiological Effects of Gemcitabine and Capecitabine Combination Chemotherapy in Advanced Pancreatic Ductal Adenocarcinoma. Br J Cancer (2016) 114(5):510–8. 10.1038/bjc.2015.468 PMC4782200 26931369

[157] Kalinsky K Diamond JR Vahdat LT Tolaney SM Juric D O’Shaughnessy J. Sacituzumab Govitecan in Previously Treated Hormone Receptor-Positive/HER2-Negative Metastatic Breast Cancer: Final Results From a Phase I/II, Single-Arm, Basket Trial. Ann Oncol (2020) 31(12):1709–18. 10.1016/j.annonc.2020.09.004 32946924

[158] Szekely B Bossuyt V Li X Wali VB Patwardhan GA Frederick C. Immunological Differences Between Primary and Metastatic Breast Cancer. Ann Oncol (2018) 29(11):2232–9. 10.1093/annonc/mdy399 30203045

[159] Caulfield SE Davis CC Byers KF. Olaparib: A Novel Therapy for Metastatic Breast Cancer in Patients With a BRCA1/2 Mutation. J Adv Pract Oncol (2019) 10(2):167–74. 10.6004/jadpro.2019.10.2.6 PMC6750920 31538027

[160] Hoy SM. Talazoparib: First Global Approval. Drugs (2018) 78(18):1939–46. 10.1007/s40265-018-1026-z 30506138

[161] Lord CJ Ashworth A. PARP Inhibitors: Synthetic Lethality in the Clinic. Science (2017) 355(6330):1152–8. 10.1126/science.aam7344 PMC6175050 28302823

[162] Ding L Kim HJ Wang Q Kearns M Jiang T Ohlson CE. Parp Inhibition Elicits STING-Dependent Antitumor Immunity in Brca1-Deficient Ovarian Cancer. Cell Rep (2018) 25(11):2972–2980 e5. 10.1016/j.celrep.2018.11.054 30540933 PMC6366450

[163] Pantelidou C Sonzogni O De Oliveria Taveira M Mehta AK Kothari A Wang D. Parp Inhibitor Efficacy Depends on CD8(+) T-Cell Recruitment Via Intratumoral Sting Pathway Activation in BRCA-Deficient Models of Triple-Negative Breast Cancer. Cancer Discovery (2019) 9(6):722–37. 10.1158/2159-8290.CD-18-1218 PMC6548644 31015319

[164] Jiao S Xia W Yamaguchi H Wei Y Chen MK Hsu JM. Parp Inhibitor Upregulates PD-L1 Expression and Enhances Cancer-Associated Immunosuppression. Clin Cancer Res (2017) 23(14):3711–20. 10.1158/1078-0432.CCR-16-3215 PMC5511572 28167507

[165] Domchek SM Postel-Vinay S Im SA Park YH Delord JP Italiano A. Olaparib and Durvalumab in Patients With Germline BRCA-mutated Metastatic Breast Cancer (MEDIOLA): An Open-Label, Multicentre, Phase 1/2, Basket Study. Lancet Oncol (2020) 21(9):1155–64. 10.1016/S1470-2045(20)30324-7 32771088

[166] Pusztai L Han HS Yau C Wolf D Wallace AM Shatsky R. Abstract CT011: Evaluation of Durvalumab in Combination With Olaparib and Paclitaxel in High-Risk HER2 Negative Stage II/III Breast Cancer: Results From the I-SPY 2 Trial. Cancer Res (2020) 80(16 Supplement):CT011–1. 10.1158/1538-7445.Am2020-ct011

[167] Mehta AK Cheney EM Hartl CA Pantelidou C Oliwa M Castrillon JA. Targeting Immunosuppressive Macrophages Overcomes PARP Inhibitor Resistance in BRCA1-associated Triple-Negative Breast Cancer. Nat Cancer (2020) 2(1):66–82. 10.1038/s43018-020-00148-7 33738458 PMC7963404

[168] Eggersmann TK Degenhardt T Gluz O Wuerstlein R Harbeck N. Cdk4/6 Inhibitors Expand the Therapeutic Options in Breast Cancer: Palbociclib, Ribociclib and Abemaciclib. BioDrugs (2019) 33(2):125–35. 10.1007/s40259-019-00337-6 30847853

[169] Schoninger SF Blain SW. The Ongoing Search for Biomarkers of CDK4/6 Inhibitor Responsiveness in Breast Cancer. Mol Cancer Ther (2020) 19(1):3–12. 10.1158/1535-7163.MCT-19-0253 31909732 PMC6951437

[170] Goel S DeCristo MJ Watt AC BrinJones H Sceneay J Li BB. CDK4/6 Inhibition Triggers Anti-Tumour Immunity. Nature (2017) 548(7668):471–5. 10.1038/nature23465 PMC5570667 28813415

[171] Zhang J Bu X Wang H Zhu Y Geng Y Nihira NT. Cyclin D-CDK4 Kinase Destabilizes PD-L1 Via Cullin 3-SPOP to Control Cancer Immune Surveillance. Nature (2018) 553(7686):91–5. 10.1038/nature25015 PMC5754234 29160310

[172] Schaer DA Beckmann RP Dempsey JA Huber L Forest A Amaladas N. The CDK4/6 Inhibitor Abemaciclib Induces a T Cell Inflamed Tumor Microenvironment and Enhances the Efficacy of PD-L1 Checkpoint Blockade. Cell Rep (2018) 22(11):2978–94. 10.1016/j.celrep.2018.02.053 29539425

[173] Rugo HS Kabos P Beck JT Chisamore MJ Hossain A Chen Y. A Phase Ib Study of Abemaciclib in Combination With Pembrolizumab for Patients With Hormone Receptor Positive (HR+), Human Epidermal Growth Factor Receptor 2 Negative (HER2-) Locally Advanced or Metastatic Breast Cancer (MBC) (NCT02779751): Interim Results. J Clin Oncol (2020) 38(15_suppl):1051–1. 10.1200/JCO.2020.38.15_suppl.1051

[174] Dickler MN Tolaney SM Rugo HS Cortes J Dieras V Patt D. Monarch 1, A Phase II Study of Abemaciclib, a CDK4 and CDK6 Inhibitor, as a Single Agent, in Patients With Refractory Hr(+)/Her2(-) Metastatic Breast Cancer. Clin Cancer Res (2017) 23(17):5218–24. 10.1158/1078-0432.CCR-17-0754 PMC5581697 28533223

[175] Rugo HS Beck JT Jerusalem G Wildiers H Kabos P Chisamore M. Abstract Ct108: A Phase 1b Study of Abemaciclib in Combination With Pembrolizumab for Patients (Pts) With Hormone Receptor Positive (HR+), Human Epidermal Growth Factor Receptor 2 Negative (HER2-) Metastatic Breast Cancer (mBC) (Nct02779751): Preliminary Results. Cancer Res (2020) 80(16 Supplement):CT108–8. 10.1158/1538-7445.Am2020-ct108

[176] Goetz MP Toi M Campone M Sohn J Paluch-Shimon S Huober J. Monarch 3: Abemaciclib as Initial Therapy for Advanced Breast Cancer. J Clin Oncol (2017) 35(32):3638–46. 10.1200/JCO.2017.75.6155 28968163

[177] Horton JK Jagsi R Woodward WA Ho A. Breast Cancer Biology: Clinical Implications for Breast Radiation Therapy. Int J Radiat Oncol Biol Phys (2018) 100(1):23–37. 10.1016/j.ijrobp.2017.08.025 29254776

[178] Demaria S Ng B Devitt ML Babb JS Kawashima N Liebes L. Ionizing Radiation Inhibition of Distant Untreated Tumors (Abscopal Effect) is Immune Mediated. Int J Radiat Oncol Biol Phys (2004) 58(3):862–70. 10.1016/j.ijrobp.2003.09.012 14967443

[179] McArthur HL Barker CA Gucalp A Lebron-Zapata L Wen YH Kallman C. A Phase II, Single Arm Study Assessing the Efficacy of Pembrolizumab (Pembro) Plus Radiotherapy (RT) in Metastatic Triple Negative Breast Cancer (Mtnbc). J Clin Oncol (2018) 36(15_suppl):1017–7. 10.1200/JCO.2018.36.15_suppl.1017

[180] Barroso-Sousa R Krop IE Trippa L Tan-Wasielewski Z Li T Osmani W. A Phase II Study of Pembrolizumab in Combination With Palliative Radiotherapy (RT) for Hormone Receptor-Positive (HR+) Metastatic Breast Cancer (MBC). J Clin Oncol (2019) 37(15_suppl):1047–7. 10.1200/JCO.2019.37.15_suppl.1047 32113750

[181] Ho AY Barker CA Arnold BB Powell SN Hu ZI Gucalp A. A Phase 2 Clinical Trialassessing Theefficacy and Safety of Pembrolizumab and Radiotherapy in Patients With Metastatic Triple-Negative Breast Cancer. Cancer (2020) 126(4):850–60. 10.1002/cncr.32599 31747077

[182] Pardoll DM. The Blockade of Immune Checkpoints in Cancer Immunotherapy. Nat Rev Cancer (2012) 12(4):252–64. 10.1038/nrc3239 PMC4856023 22437870

[183] Acharya N Sabatos-Peyton C Anderson AC. Tim-3 Finds its Place in the Cancer Immunotherapy Landscape. J Immunother Cancer (2020) 8(1):e000911. 10.1136/jitc-2020-000911 32601081 PMC7326247

[184] Chauvin J-M Zarour HM. TIGIT in Cancer Immunotherapy. J ImmunoTher Cancer (2020) 8(2):e000957. 10.1136/jitc-2020-000957 32900861 PMC7477968

[185] Munn DH Mellor AL. IDO in the Tumor Microenvironment: Inflammation, Counter-Regulation, and Tolerance. Trends Immunol (2016) 37(3):193–207. 10.1016/j.it.2016.01.002 26839260 PMC4916957

[186] Workman CJ Vignali DA. Negative Regulation of T Cell Homeostasis by Lymphocyte Activation Gene-3 (CD223). J Immunol (2005) 174(2):688–95. 10.4049/jimmunol.174.2.688 15634887

[187] Anurag M Zhu M Huang C Vasaikar S Wang J Hoog J. Immune Checkpoint Profiles in Luminal B Breast Cancer (Alliance). J Natl Cancer Inst (2020) 112(7):737–46. 10.1093/jnci/djz213 PMC7805027 31665365

[188] Bendell JC Varghese AM Hyman DM Bauer TM Pant S Callies S. A First-in-Human Phase 1 Study of LY3023414, an Oral PI3K/Mtor Dual Inhibitor, in Patients With Advanced Cancer. Clin Cancer Res (2018) 24(14):3253–62. 10.1158/1078-0432.CCR-17-3421 29636360

[189] Gibney GT Hamid O Lutzky J Olszanski AJ Mitchell TC Gajewski TF. Phase 1/2 Study of Epacadostat in Combination With Ipilimumab in Patients With Unresectable or Metastatic Melanoma. J Immunother Cancer (2019) 7(1):80. 10.1186/s40425-019-0562-8 30894212 PMC6425606

[190] Yue EW Sparks R Polam P Modi D Douty B Wayland B. INCB24360 (Epacadostat), a Highly Potent and Selective Indoleamine-2,3-dioxygenase 1 (Ido1) Inhibitor for Immuno-Oncology. ACS Med Chem Lett (2017) 8(5):486–91. 10.1021/acsmedchemlett.6b00391 PMC5430407 28523098

[191] Wu L Lv C Su Y Li C Zhang H Zhao X. Expression of Programmed Death-1 (PD-1) and its Ligand PD-L1 is Upregulated in Endometriosis and Promoted by 17beta-Estradiol. Gynecol Endocrinol (2019) 35(3):251–6. 10.1080/09513590.2018.1519787 30325236

[192] Polanczyk MJ Hopke C Vandenbark AA Offner H. Treg Suppressive Activity Involves Estrogen-Dependent Expression of Programmed Death-1 (PD-1). Int Immunol (2007) 19(3):337–43. 10.1093/intimm/dxl151 17267414

[193] Shen Z Rodriguez-Garcia M Patel MV Barr FD Wira CR. Menopausal Status Influences the Expression of Programmed Death (PD)-1 and its Ligand PD-L1 on Immune Cells From the Human Female Reproductive Tract. Am J Reprod Immunol (2016) 76(2):118–25. 10.1111/aji.12532 PMC4942364 27321759

[194] Yang L Huang F Mei J Wang X Zhang Q Wang H. Posttranscriptional Control of PD-L1 Expression by 17beta-Estradiol Via PI3K/Akt Signaling Pathway in ERalpha-Positive Cancer Cell Lines. Int J Gynecol Cancer (2017) 27(2):196–205. 10.1097/IGC.0000000000000875 27870715 PMC5258765

[195] Tuohy VK Jaini R Johnson JM Loya MG Wilk D Downs-Kelly E. Targeted Vaccination Against Human alpha-Lactalbumin for Immunotherapy and Primary Immunoprevention of Triple Negative Breast Cancer. Cancers (Basel) (2016) 8(6):56. 10.3390/cancers8060056 PMC4931621 27322324

[196] Jaini R Loya MG Eng C. Immunotherapeutic Target Expression on Breast Tumors can be Amplified by Hormone Receptor Antagonism: A Novel Strategy for Enhancing Efficacy of Targeted Immunotherapy. Oncotarget (2017) 8(20):32536–49. 10.18632/oncotarget.15812 PMC5464807 28430646

[197] Marquez-Garban DC Deng G Comin-Anduix B Garcia AJ Xing Y Chen HW. Antiestrogens in Combination With Immune Checkpoint Inhibitors in Breast Cancer Immunotherapy. J Steroid Biochem Mol Biol (2019) 193:105415. 10.1016/j.jsbmb.2019.105415 31226312 PMC6903431

[198] Tsao LC Crosby EJ Trotter TN Agarwal P Hwang BJ Acharya C. CD47 Blockade Augmentation of Trastuzumab Antitumor Efficacy Dependent on Antibody-Dependent Cellular Phagocytosis. JCI Insight (2019) 4(24):e131882. 10.1172/jci.insight.131882 PMC6975273 31689243

[199] Cook KL Soto-Pantoja DR Clarke PA Cruz MI Zwart A Warri A. Endoplasmic Reticulum Stress Protein Grp78 Modulates Lipid Metabolism to Control Drug Sensitivity and Antitumor Immunity in Breast Cancer. Cancer Res (2016) 76(19):5657–70. 10.1158/0008-5472.CAN-15-2616 PMC5117832 27698188

[200] Cook KL Soto-Pantoja DR. “Upregulation” of CD47 by the Endoplasmic Reticulum Stress Pathway Controls Anti-Tumor Immune Responses. Biomark Res (2017) 5:26. 10.1186/s40364-017-0105-8 28815041 PMC5557514

[201] Zhang H Lu H Xiang L Bullen JW Zhang C Samanta D. HIF-1 Regulates CD47 Expression in Breast Cancer Cells to Promote Evasion of Phagocytosis and Maintenance of Cancer Stem Cells. Proc Natl Acad Sci USA (2015) 112(45):E6215–23. 10.1073/pnas.1520032112 PMC4653179 26512116

[202] Betancur PA Abraham BJ Yiu YY Willingham SB Khameneh F Zarnegar M. A CD47-associated Super-Enhancer Links Pro-Inflammatory Signalling to CD47 Upregulation in Breast Cancer. Nat Commun (2017) 8:14802. 10.1038/ncomms14802 28378740 PMC5382276

[203] Jaiswal S Jamieson CH Pang WW Park CY Chao MP Majeti R. CD47 is Upregulated on Circulating Hematopoietic Stem Cells and Leukemia Cells to Avoid Phagocytosis. Cell (2009) 138(2):271–85. 10.1016/j.cell.2009.05.046 PMC2775564 19632178

[204] Sikic BI Lakhani N Patnaik A Shah SA Chandana SR Rasco D. First-in-Human, First-in-Class Phase I Trial of the Anti-CD47 Antibody Hu5F9-G4 in Patients With Advanced Cancers. J Clin Oncol (2019) 37(12):946–53. 10.1200/JCO.18.02018 PMC7186585 30811285

[205] Qiu SQ Waaijer SJH Zwager MC de Vries EGE van der Vegt B Schroder CP. Tumor-Associated Macrophages in Breast Cancer: Innocent Bystander or Important Player? Cancer Treat Rev (2018) 70:178–89. 10.1016/j.ctrv.2018.08.010 30227299

[206] Garvin S Vikhe Patil E Arnesson LG Oda H Hedayati E Lindstrom A. Differences in Intra-Tumoral Macrophage Infiltration and Radiotherapy Response Among Intrinsic Subtypes in pT1-T2 Breast Cancers Treated With Breast-Conserving Surgery. Virchows Arch (2019) 475(2):151–62. 10.1007/s00428-019-02563-3 PMC6647441 30915533

[207] Gwak JM Jang MH Kim DI Seo AN Park SY. Prognostic Value of Tumor-Associated Macrophages According to Histologic Locations and Hormone Receptor Status in Breast Cancer. PloS One (2015) 10(4):e0125728. 10.1371/journal.pone.0125728 25884955 PMC4401667

[208] Lofdahl B Ahlin C Holmqvist M Holmberg L Zhou W Fjallskog ML. Inflammatory Cells in Node-Negative Breast Cancer. Acta Oncol (2012) 51(5):680–6. 10.3109/0284186X.2011.652737 22268578

[209] Mahmoud SM Lee AH Paish EC Macmillan RD Ellis IO Green AR. Tumour-Infiltrating Macrophages and Clinical Outcome in Breast Cancer. J Clin Pathol (2012) 65(2):159–63. 10.1136/jclinpath-2011-200355 22049225

[210] Yuan J He H Chen C Wu J Rao J Yan H. Combined High Expression of CD47 and CD68 is a Novel Prognostic Factor for Breast Cancer Patients. Cancer Cell Int (2019) 19:238. 10.1186/s12935-019-0957-0 31528120 PMC6737685

[211] Zhang Y Cheng S Zhang M Zhen L Pang D Zhang Q. High-Infiltration of Tumor-Associated Macrophages Predicts Unfavorable Clinical Outcome for Node-Negative Breast Cancer. PloS One (2013) 8(9):e76147. 10.1371/journal.pone.0076147 24098773 PMC3786995

[212] Xuan QJ Wang JX Nanding A Wang ZP Liu H Lian X. Tumor-Associated Macrophages are Correlated With Tamoxifen Resistance in the Postmenopausal Breast Cancer Patients. Pathol Oncol Res (2014) 20(3):619–24. 10.1007/s12253-013-9740-z 24414992

[213] Vonderheide RH. CD47 Blockade as Another Immune Checkpoint Therapy for Cancer. Nat Med (2015) 21(10):1122–3. 10.1038/nm.3965 26444633

[214] Thorsson V Gibbs DL Brown SD Wolf D Bortone DS Ou Yang TH. The Immune Landscape of Cancer. Immunity (2018) 48(4):812–830 e14. 10.1016/j.immuni.2018.03.023 29628290 PMC5982584

[215] Newman AM Liu CL Green MR Gentles AJ Feng W Xu Y. Robust Enumeration of Cell Subsets From Tissue Expression Profiles. Nat Methods (2015) 12(5):453–7. 10.1038/nmeth.3337 PMC4739640 25822800

[216] Zhang SC Hu ZQ Long JH Zhu GM Wang Y Jia Y. Clinical Implications of Tumor-Infiltrating Immune Cells in Breast Cancer. J Cancer (2019) 10(24):6175–84. 10.7150/jca.35901 PMC6856577 31762828

[217] Cassetta L Fragkogianni S Sims AH Swierczak A Forrester LM Zhang H. Human Tumor-Associated Macrophage and Monocyte Transcriptional Landscapes Reveal Cancer-Specific Reprogramming, Biomarkers, and Therapeutic Targets. Cancer Cell (2019) 35(4):588–602.e10. 10.1016/j.ccell.2019.02.009 30930117 PMC6472943

[218] Edgar R Domrachev M Lash AE. Gene Expression Omnibus: NCBI Gene Expression and Hybridization Array Data Repository. Nucleic Acids Res (2002) 30(1):207–10. 10.1093/nar/30.1.207 PMC99122 11752295

[219] Desmedt C Haibe-Kains B Wirapati P Buyse M Larsimont D Bontempi G. Biological Processes Associated With Breast Cancer Clinical Outcome Depend on the Molecular Subtypes. Clin Cancer Res (2008) 14(16):5158–65. 10.1158/1078-0432.CCR-07-4756 18698033

[220] McKinney EF Lyons PA Carr EJ Hollis JL Jayne DR Willcocks LC. A Cd8+ T Cell Transcription Signature Predicts Prognosis in Autoimmune Disease. Nat Med (2010) 16(5):586–91, 1p following 591. 10.1038/nm.2130 20400961 PMC3504359

[221] Perez EA Thompson EA Ballman KV Anderson SK Asmann YW Kalari KR. Genomic Analysis Reveals That Immune Function Genes are Strongly Linked to Clinical Outcome in the North Central Cancer Treatment Group N9831 Adjuvant Trastuzumab Trial. J Clin Oncol (2015) 33(7):701–8. 10.1200/JCO.2014.57.6298 PMC4334774 25605861

[222] Teschendorff AE Miremadi A Pinder SE Ellis IO Caldas C. An Immune Response Gene Expression Module Identifies a Good Prognosis Subtype in Estrogen Receptor Negative Breast Cancer. Genome Biol (2007) 8(8):R157. 10.1186/gb-2007-8-8-r157 17683518 PMC2374988

[223] Quigley DA Tahiri A Luders T Riis MH Balmain A Borresen-Dale AL. Age, Estrogen, and Immune Response in Breast Adenocarcinoma and Adjacent Normal Tissue. Oncoimmunology (2017) 6(11):e1356142. 10.1080/2162402X.2017.1356142 29147603 PMC5674948

[224] Azizi E Carr AJ Plitas G Cornish AE Konopacki C Prabhakaran S. Single-Cell Map of Diverse Immune Phenotypes in the Breast Tumor Microenvironment. Cell (2018) 174(5):1293–1308 e36. 10.1016/j.cell.2018.05.060 29961579 PMC6348010

[225] Wagner J Rapsomaniki MA Chevrier S Anzeneder T Langwieder C Dykgers A. A Single-Cell Atlas of the Tumor and Immune Ecosystem of Human Breast Cancer. Cell (2019) 177(5):1330–1345 e18. 10.1016/j.cell.2019.03.005 30982598 PMC6526772

[226] Stack EC Wang C Roman KA Hoyt CC. Multiplexed Immunohistochemistry, Imaging, and Quantitation: A Review, With an Assessment of Tyramide Signal Amplification, Multispectral Imaging and Multiplex Analysis. Methods (2014) 70(1):46–58. 10.1016/j.ymeth.2014.08.016 25242720

[227] Lin JR Izar B Wang S Yapp C Mei S Shah PM. Highly Multiplexed Immunofluorescence Imaging of Human Tissues and Tumors Using t-CyCIF and Conventional Optical Microscopes. Elife (2018) 7:e31657. 10.7554/eLife.31657 29993362 PMC6075866

[228] Goltsev Y Samusik N Kennedy-Darling J Bhate S Hale M Vazquez G. Deep Profiling of Mouse Splenic Architecture With CODEX Multiplexed Imaging. Cell (2018) 174(4):968–981 e15. 10.1016/j.cell.2018.07.010 30078711 PMC6086938

[229] Ptacek J Locke D Finck R Cvijic ME Li Z Tarolli JG. Multiplexed Ion Beam Imaging (MIBI) for Characterization of the Tumor Microenvironment Across Tumor Types. Lab Invest (2020) 100(8):1111–23. 10.1038/s41374-020-0417-4 32203152

[230] Giesen C Wang HA Schapiro D Zivanovic N Jacobs A Hattendorf B. Highly Multiplexed Imaging of Tumor Tissues With Subcellular Resolution by Mass Cytometry. Nat Methods (2014) 11(4):417–22. 10.1038/nmeth.2869 24584193

[231] Tsujikawa T Kumar S Borkar RN Azimi V Thibault G Chang YH. Quantitative Multiplex Immunohistochemistry Reveals Myeloid-Inflamed Tumor-Immune Complexity Associated With Poor Prognosis. Cell Rep (2017) 19(1):203–17. 10.1016/j.celrep.2017.03.037 PMC5564306 28380359

[232] Tsujikawa T Thibault G Azimi V Sivagnanam S Banik G Means C. Robust Cell Detection and Segmentation for Image Cytometry Reveal Th17 Cell Heterogeneity. Cytometry A (2019) 95(4):389–98. 10.1002/cyto.a.23726 PMC6461524 30714674

[233] Young G Hundt N Cole D Fineberg A Andrecka J Tyler A. Quantitative Mass Imaging of Single Biological Macromolecules. Science (2018) 360(6387):423–7. 10.1126/science.aar5839 PMC6103225 29700264

[234] Ali HR Jackson HW Zanotelli VRT Danenberg E Fischer JR Bardwell H. Imaging Mass Cytometry and Multiplatform Genomics Define the Phenogenomic Landscape of Breast Cancer. Nat Cancer (2020) 1(2):163–75. 10.1038/s43018-020-0026-6 35122013

[235] Jackson HW Fischer JR Zanotelli VRT Ali HR Mechera R Soysal SD. The Single-Cell Pathology Landscape of Breast Cancer. Nature (2020) 578(7796):615–20. 10.1038/s41586-019-1876-x 31959985

[236] Kalluri R. The Biology and Function of Fibroblasts in Cancer. Nat Rev Cancer (2016) 16(9):582–98. 10.1038/nrc.2016.73 27550820

[237] Patten DK Corleone G Gyorffy B Perone Y Slaven N Barozzi I. Enhancer Mapping Uncovers Phenotypic Heterogeneity and Evolution in Patients With Luminal Breast Cancer. Nat Med (2018) 24(9):1469–80. 10.1038/s41591-018-0091-x PMC6130800 30038216

